# Suicides, suicide attempts and suicidal ideation among children and young people exposed to war: a scoping review

**DOI:** 10.1136/bmjopen-2025-103126

**Published:** 2026-06-04

**Authors:** Sanju Silwal, Minja Westerlund, Wan Mohd Azam Wan Mohd Yunus, Saara Jaakola-Siimes, Anat Brunstein Klomek, Andre Sourander

**Affiliations:** 1 Department of Child Psychiatry, Research Centre for Child Psychiatry, University of Turku, Turku, Finland; 2 INVEST Flagship, University of Turku, Turku, Finland; 3 Faculty of Social Sciences and Humanities, Universiti Teknologi Malaysia, Johor, Malaysia; 4 TYKS Turku University Hospital, Turku, Finland; 5 Baruch Ivcher School of Psychology, Reichman University, Herzliya, Israel

**Keywords:** Systematic Review, Adolescent, Suicide & self-harm, Child

## Abstract

**Abstract:**

**Objectives:**

Worldwide, billions of children and young people live in areas affected by war. Suicide remains one of the three leading causes of death worldwide among people aged 15–29 years. However, little is known about the effect of war on suicidal behaviours in this group. This review aims to assess suicides, suicide attempts and suicidal ideation among children and young people exposed to war or armed conflict.

**Design:**

A scoping review of studies was conducted using Web of Science, PubMed, Embase and PsycINFO databases from their inception to 18 November 2025, without any restrictions on geographical location. We included only observational studies with full-text, peer-reviewed English articles reporting any suicides, suicide attempts and suicidal ideation of children and young people aged 0–24 years exposed to war. The quality of the included articles was assessed using the Quality Assessment with Diverse Studies. The protocol of the review was registered with the Open Science Framework on 29 March 2022 (https://osf.io/7kszh/).

**Results:**

Of the 3229 articles retrieved, 37 studies were eligible for review, providing data from 24 countries and covering a period of almost a hundred years (1921–2025). Most studies (>20) focused on conflicts ongoing during or until the 2000s, whereas only three focused on World War II. The reported outcomes were suicides (n=9), suicide attempts (n=15) and suicidal ideation (n=21). Included studies spanned six continents, from Latin America (n=5, Colombia only) to Europe (n=10). We assessed the suicide rates during and after wars. There was some evidence of a decrease in suicide rates during war, but no clear trend in suicide rates post-war was observed. The prevalence rates of suicide attempts and suicidal ideation varied widely, without uniformity in the definitions used. War-related trauma, mental health problems, substance abuse and exposure to suicide or suicide attempts were identified as risk factors, while protective factors included family and social support.

**Conclusions:**

There is a need for more methodologically consistent and rigorous research on suicidal thoughts and behaviours in children and young people exposed to war or armed conflicts. Future research should identify mediator/moderating factors influencing suicidal behaviours and their links to mental health.

**STRENGTHS AND LIMITATIONS OF THIS STUDY**

- To our knowledge, this is the first scoping review on suicides, suicide attempts and suicidal ideation among children and young people exposed to war or armed conflicts.
- Our review included 37 observational studies from 24 countries, covering a period of a hundred years (1921–2025).
- This review only included English-language articles.
- Assessment tools were heterogeneous, limiting our ability to draw firm conclusions across studies and generalisability.
- Under-representation of some major conflict zones, namely Africa or the Middle East, where larger populations of children and young adults are affected by ongoing wars.

## Introduction

 In modern warfare, civilian deaths account for an estimated 13%–87% of all casualties,^1^ and in nearly every war, children are among the main victims.^2^ Civilians in conflict settings, among them minors,^3^ often lose their lives to direct war violence; however, the detrimental effects of armed conflict also extend beyond military attacks.^4^ War causes immense destruction across most aspects of life, from infrastructure, institutions and environment to health and the social fabric of communities. Children and adolescents are especially impacted by war, given their need to develop within a safe and predictable environment.^5 6^ Indeed, minors exposed to war have an elevated risk of developing psychopathologies.^5^ This is especially true for adolescents, who are better able to understand the full impact of war and are often more exposed to it.^7^ Importantly, human reactions to collective catastrophes such as wars result from complex interactions involving not only psychological but also biological, cultural, social and historical factors.^8^ Therefore, adverse emotional reactions to armed conflict should be interpreted in relation to a larger framework of social determinants,^9^ and they should not be reduced to a deterministic and pathologising psychiatric label.^8^ Undeniably, however, armed conflict does produce detrimental psychological harm, and amidst the profound suffering that war entails, some do ultimately resort to suicidality.^10^

Suicides and suicidal behaviours are global public health concerns. Suicide remains one of the three leading causes of death worldwide among people aged 15–29 years.^11^ Suicide attempts and suicidal ideation are antecedents of suicides in children and young people.^12^ Theories describing the transition from suicidal ideation to action have originally been developed among adults, but recent studies have shown some support among adolescents as well.^13^ According to one such theory, the Three-Step Theory (3ST),^14^ suicidal ideation starts in an existence marked by, most often, psychological or emotional pain and without hope for improvement—conditions that are characteristically prevalent in war settings. The experienced pain, which emerges from an interplay of other factors, such as mental health disorders, temperament and experiences (eg, losses), decreases the individual’s desire to live. Notably, children and adolescents have not reached full cognitive maturity, which further affects how they interpret losses and threats related to wars.^10^

The next step in developing suicidal ideation involves disrupted connectedness, usually to other people, but also to roles, interests or something else perceived as meaningful. In wartime, social, educational and economic disruptions, as well as physical and cultural displacements, are prevalent, which in some circumstances may undermine an individual’s sense of purpose and decrease connectedness to life. Lastly, according to the 3ST, dispositional (eg, pain sensitivity), acquired (ie, habituation to aversive experiences) and practical factors (eg, access to lethal means) determine whether the ideation translates into suicide attempts.

In 2024, the number of armed conflicts rose worldwide, with 61 active conflicts involving at least one state.^15^ Of these, 11 escalated to the level of war, defined as a conflict resulting in at least 1000 battle-related deaths in a year. While certain efforts have been made throughout human history to protect civilians in war, and increasingly so since World War II, advancements in weapon technology and the growing number of conflicts, on the other hand, pose a greater threat to civilian lives and well-being.^16 17^ As a result of violent conflicts globally, today, 47.2 million children are displaced^18^ and are disproportionately affected by armed conflicts.^2 3^ Previous reviews have highlighted the damaging effect of war on children’s and young people’s mental health,^19 20^ but only one review has reported suicide attempts and suicidal ideation among young refugees in European countries.^21^ To our knowledge, no review has specifically examined suicides, suicide attempts and suicidal ideations among children and young people exposed to armed conflict. Even though civilian populations’ experience of war differs across war settings,^17^ considering the number of children and young people exposed to the terror of war worldwide, suicidality may be a significant public health problem among young and vulnerable war-exposed populations.

This scoping review aims to map and describe available epidemiological studies examining suicides, suicide attempts and suicidal ideations among children and young people exposed to war or armed conflicts. We also aim to identify trauma exposures and risk and protective factors associated with the outcomes. We include all populations aged 0–24 years as per the WHO definition of young people,^22^ while acknowledging that the notion of childhood and, with that, the social expectations for children’s roles and responsibilities differ considerably across social contexts^2^ and historical periods.^23^

## Methods

### Search strategy and selection criteria

The scoping review was conducted in accordance with the Preferred Reporting Items for Systematic Reviews and Meta-Analyses extension for scoping reviews (PRISMA-ScR).^24^ The protocol of the review was registered with the Open Science Framework on 29 March 2022 (https://osf.io/7kszh/). Studies were identified through comprehensive searches of electronic databases and other sources. The following electronic databases were used: Web of Science, PubMed, Embase and PsycINFO. Keywords for searching titles and abstracts were developed with guidance from librarians. Three sets of keywords were used and modified for each database: (1) ‘War’ OR ‘Armed Conflicts’; (2) ‘Suicide’ OR ‘Suicide attempts’ OR ‘Suicidal Ideation’ and (3) ‘Child’ OR ‘Adolescent’ OR ‘Young people’. A detailed search strategy is available in online supplemental material 1. Searches were conducted for studies published up to 18 November 2025. Searches were not limited by geographic location. Only full-text, English-language, peer-reviewed articles were included. The reference lists of relevant articles and reviews were also screened to identify potential additional studies. The inclusion criteria for the review were (1) observational studies, including retrospective, prospective, cross-sectional, longitudinal and case-control studies; (2) studies reporting any suicides, suicide attempts and suicidal ideation of children and young people aged 0–24 years exposed to war or armed conflict, with a flexibility of ±1 year (studies, including participants above this age group, were included if results were reported separately). The WHO defines young people as individuals aged 10–24 years,^22^ and the definition of young people fits well with the period of ‘adolescence’ defined by Sawyer *et al*^25^; (3) peer-reviewed studies and (4) English-language studies. Studies were excluded if: (1) they were conference abstracts, case reports, reviews, only qualitative studies or case series; (2) they included participants aged more than 24 years and (3) the language of the study was not English.

#### Study selection

Three reviewers (SS, WMAWMY and SJ-S) independently scanned the titles and abstracts of the papers. Each article was independently assessed by at least two reviewers. When more information was needed, the full text was obtained to determine the study’s final eligibility. In case of any disagreements, final decisions were made after discussing with senior researchers (MW and AS). The authors were contacted for additional information, if needed (see online supplemental table 2).

#### Data extraction

A checkbox table was used to depict comparisons between the included studies. Key information (where available) was extracted into an Excel spreadsheet: first author, year, country/region, study design, study period, type of conflict, sample size, age, data source, trauma exposure measurement, outcome measures, statistical analysis, key findings, subgroup analysis findings, risk and protective factors, other findings and the reviewer’s personal comments. The data extraction was conducted by SS, WMAWMY and SJ-S, and the accuracy of the data extraction was cross-checked. For studies identified in our search, at least two reviewers independently extracted the relevant data. Discrepancies were resolved in consultation with a third reviewer (AS). We contacted the corresponding authors to obtain missing information and sent reminder emails 1 week later.

#### Data synthesis

Three categories of outcomes were assessed in the study: suicides, suicide attempts and suicidal ideation. Suicides referred to suicide deaths caused by self-directed injurious behaviour with the intent to die.^26^ Suicide attempts were defined as self-directed, potentially injurious behaviours with the intent to die. Suicidal ideation referred to thoughts of engaging in suicidal behaviour. Data were included in the review when these outcomes were measured during or after exposure to war or armed conflict. We conducted a narrative synthesis of the included studies. We organised the results of the scoping review by various geographical regions, type of conflict and key characteristics of the included studies. The results were presented based on estimates from the included studies, for example, the prevalence of suicides, suicide attempts and suicidal ideation and relevant risk and protective factors associated with the outcomes.

### Quality assessment

Each study was scored according to the Quality Assessment with Diverse Studies (QuADS)^27^ independently by three reviewers (SS, WMAWMY and SJ-S). This tool can be applied to a methodologically diverse set of studies by employing 13 items with four-point scales (0–3), or ‘not applicable’ where it is not applicable to that particular study. The mean scores for each item were then calculated to identify deficiencies in the study quality. Findings from a higher-quality paper were given more weight in the discussion of the paper.

### Patient and public involvement

Patients and/or the public were not involved in the design, conduct, reporting or dissemination plans of this research.

## Results

### Search results

**Figure 1:**
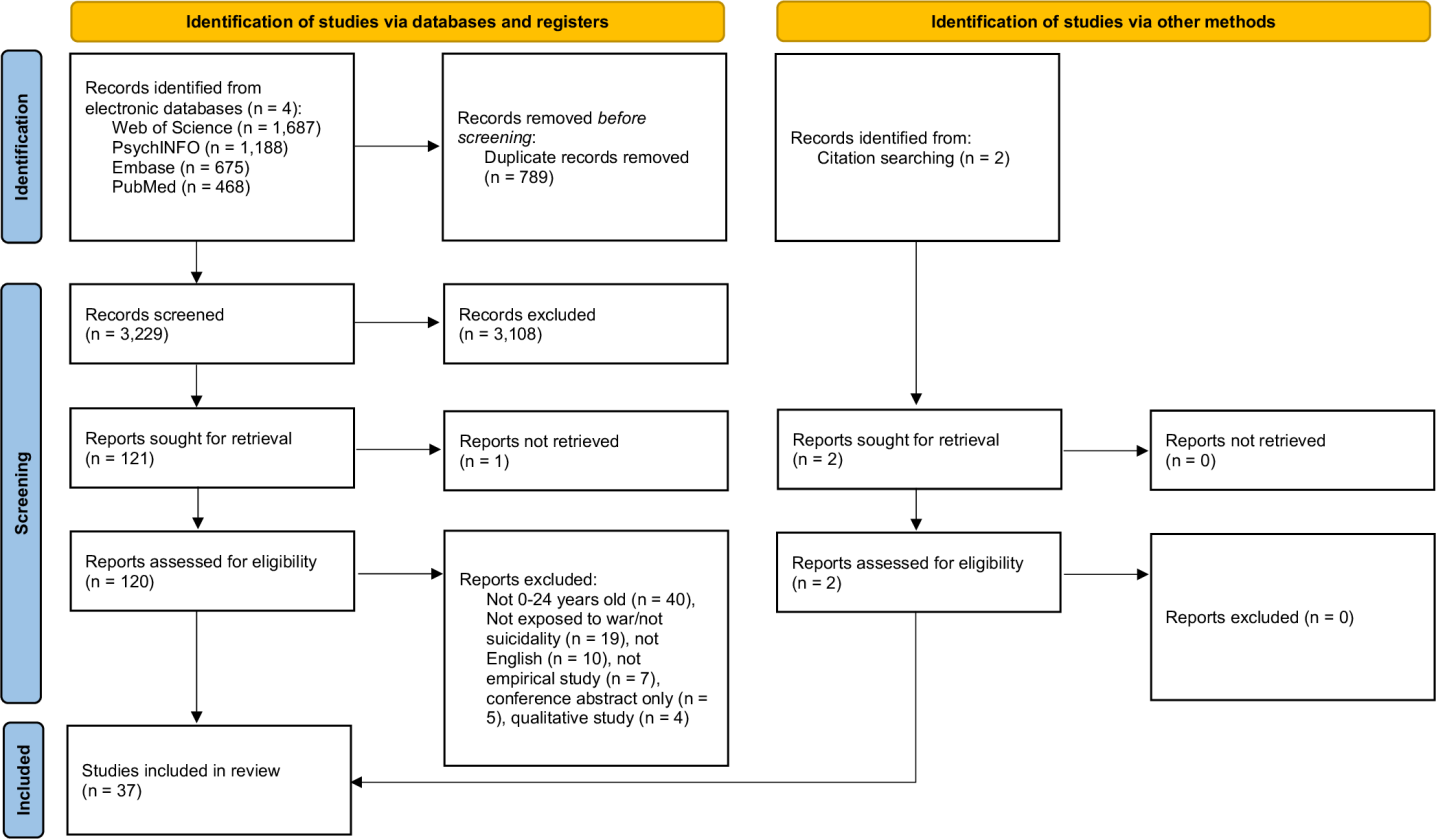
Preferred Reporting Items for Systematic Reviews and Meta-Analyses extension for scoping reviews (PRISMA-ScR) flow diagram of the study.

The initial database and manual searches identified 4022 citations, and 789 duplicates were removed. Then, the titles and abstracts of 3229 articles were screened for eligibility based on the inclusion and exclusion criteria of the review. Out of 120 full texts, 68 papers were excluded (see online supplemental material 3). Two articles were found in the reference list. Finally, 37 articles were included in this review. Figure 1 displays the PRISMA flow diagram for the screening and study selection process.

#### Characteristics of the included studies

**Figure 2:**
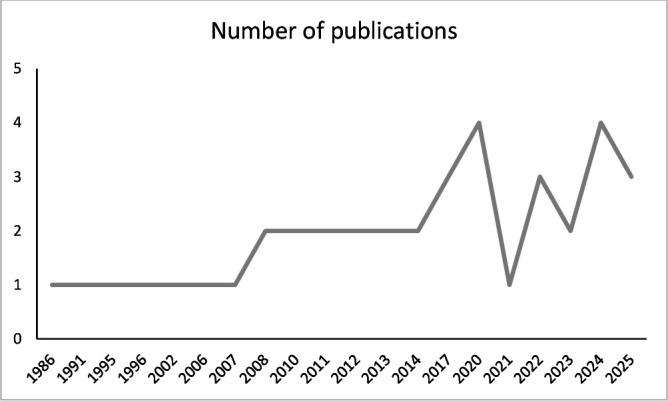
Number of publications by year.

**Figure 3:**
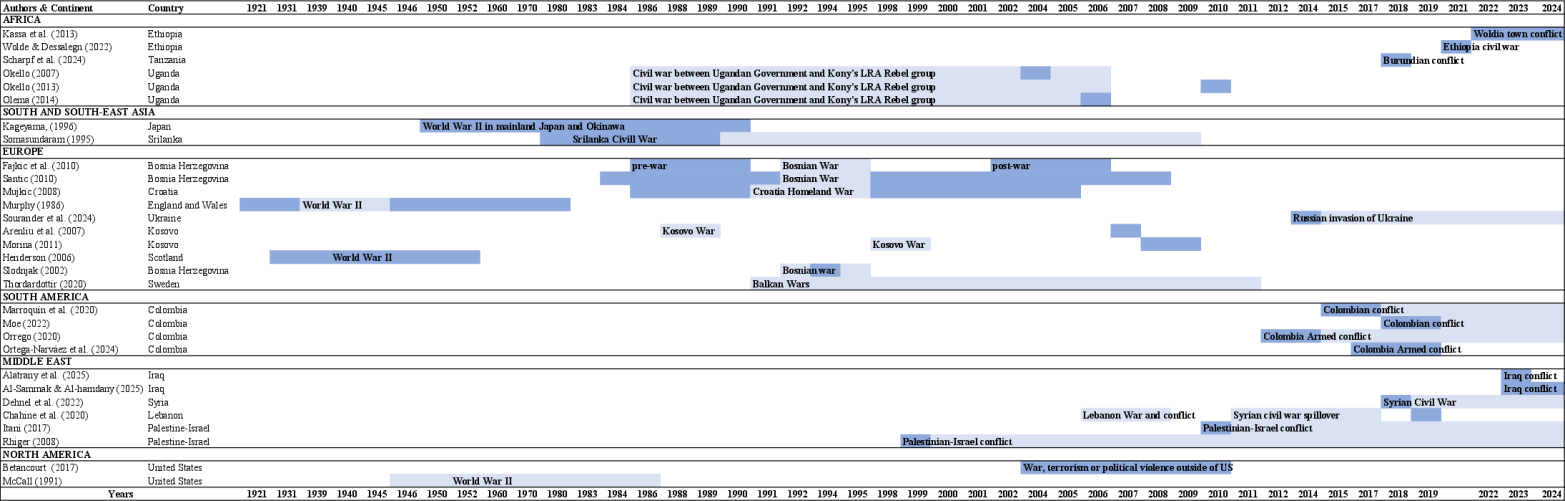
Study period (years) and type of conflict assessed in included studies. LRA, Lord's Resistance Army insurgency.

Of the 37 studies included, 28 were cross-sectional, 7 were time trends and 2 were cohort studies. The characteristics of the included studies are presented in table 1. Online supplemental tables 4 and 5 present detailed information on each study. The review included studies published between 1986 and 2025 (see figure 2). The reported outcomes were suicides (n=9), suicide attempts (n=15) and suicidal ideation (n=21), with some studies reporting more than one outcome. The studies covered suicidal outcomes exposed to wars and armed conflicts covering a period of a hundred years (1921–2025; see figure 3). The studies comprised 288 073 subjects from 24 countries: 10 from Europe, 8 from the Middle East, 7 from Africa, 5 from Latin America, 4 from East and Southeast Asia and 3 from North America. One study reported the pooled prevalence of suicide attempts and suicidal ideation from multiple countries.^28^

**Table 1:** Characteristics of the included studies

	Number of studies
Geographical locations	
Africa	7
Europe	10
Middle East	8
South and South-East Asia	4
North America	3
Latin America	5
Research design	
Cross-sectional	28
Cohort	2
Time trend	7
Population	
General population	28
Refugees	5
Displaced	2
Abducted	2
Bereaved	1
Methodology	
School-based	16
Community-based	3
Registers or records	12
Displacement camps or centres	4
Clinics	2
Studied domain	
Suicide	9
Suicide attempts	15
Suicidal ideation	21
Informants	
Self-report	16
Clinical interview	12
Coroner’s record	5

The population varied from general population (n=28), clinical (n=3), internally displaced (n=2), refugees (n=5), abducted or non-abducted children (n=2) or bereaved (n=1). These studies included participants from different settings: schools (n=16), registers or records (n=12), displacement camps or centres (n=4), community (n=3) and clinics (n=2). The number of participants ranged from 52 to 252 200, with most studies having sample sizes of less than 1000.

We used the definitions of Uppsala Conflict Data Programme,^29^ according to which armed conflict is a state-based conflict concerning the use of armed forces between two parties, of which at least one is the government of a state, resulting in at least 25 battle-related deaths in one calendar year. War is a state-based conflict resulting in at least 1000 battle-related deaths in a specific calendar year. Most common types of conflict addressed in the studies were intrastate conflicts, of which most also included instances of one-sided violence (ie, use of armed force against civilians by a government actor or formally organised group),^30^ for example, the Colombian conflict (n=5) and the civilian war between Uganda and Kony’s Lord's Resistance Army insurgency Rebel group (n=3). Interstate conflicts were addressed in studies relating to World War II and the recent Russia-Ukraine War, although some conflicts included interstate elements (eg, conflicts in Lebanon). Geographically, these interstate studies were limited to Europe and the USA, whereas studies from intrastate conflict contexts were spread across all regions covered in the current review. The nature of these conflicts, and their spreading over time, is in line with the scholarly consensus that modern wars are more often conducted within states than between states.^9^ In the past 10–20 years, studies reported suicidal outcomes from the Palestinian-Israeli conflict, Syrian Civil War, Lebanese conflict, Colombian conflict and the Russian invasion of Ukraine.

#### Quality assessment

The quality assessment of the 37 included studies was based on 13 criteria of QuADS (see online supplemental table 6). The quality of reviewed studies ranged from 19 to 38. A detailed description of qualitative assessment scores is presented in online supplemental table 6. The four highest scores were recorded for the following criteria: Criteria 3: a clear description of the research setting and target population; Criteria 4: the study design is appropriate to address the stated research aim/s; Criteria 7: the format and content of the data collection tool are appropriate to address the stated research aim/s and Criteria 11: the method of analysis was appropriate to answer the research aims. The lowest scores were recorded for Criteria 12: evidence that the research stakeholders have been considered in the research design or conduct and Criteria 10: justification for analytic method selected. Most studies did not involve stakeholders in the research design or conduct a pilot study, which influenced the design or conduct of the study.

### Outcomes

#### Suicides

Nine studies examined suicides from various wars, including World War II, the Bosnian War, the Croatian War and the Sri Lankan Civil War (see online supplemental tables 3 and 4). Three studies reported suicide rates before, during and after wars.^31^,^34^ One study reported suicide rates before and after the Bosnian War,^35^ while another compared rates before and during the Sri Lankan Civil War.^36^ Additionally, one study assessed suicide rates among immigrants exposed to the Balkan War compared with non-migrants in Sweden.^37^

Four studies focused on suicides during and after World War II. During World War II, one study from England and Wales reported a decrease in suicide rates,^38^ whereas a study from Scotland showed an increase, particularly among men.^31^ During the Croatian Homeland War, a study reported an increase in weapon-related suicide rates during the war.^33^ In the Sri Lankan Civil War, suicide rates decreased during the war compared with pre-war levels. During the Bosnian War, one study showed a decline in suicide rates 8 years after the war,^35^ while another study showed a decrease during the war but an increase afterwards.^34^

After World War II, the suicide rates decreased in Japan, followed by an increase later.^39^ In the USA, suicide rates remained stable for a few years after the war before rising sharply.^32^ Increases in weapon-related rates were reported after the Croatian Homeland War.^33^ Additionally, suicide rates were found to be higher among Balkan War migrants compared with non-war migrants.^37^ The methods used for suicides across studies included hanging, firearms, sleeping pills and drowning.^34^,^36^

#### Suicide attempts and suicidal ideation

Suicide attempts were reported in 15 studies, and suicidal ideation was assessed in 21 studies. As summarised in online supplemental tables 3 and 4, the prevalence of suicide rates varied widely across studies, ranging from 3.2% to 65.0%,^40 41^ and suicidal ideation ranged from 7.7% to 34.5%.^37 38^ Assessment tools varied widely; some studies used single items, standardised scales or International Statistical Classification of Diseases and Related Health Problems codes. The Composite International Diagnostic Interview was used in three studies,^42^,^44^ and the Mini International Neuropsychiatric Interview in two studies.^45 46^ Suicide attempt codes from registers were used in two studies.^37 47^ Elevated rates of suicide attempts and ideation were observed among displaced adolescents,^43^ bereaved children^42^ and those exposed to community violence.^48^ Gender differences were evident, with higher rates among girls compared with boys in three studies.^41 49 50^

#### Trauma exposure and risk and protective factors

Wartime traumatic events were associated with suicide attempts and suicidal ideation, with the highest odds among victims of violence, forced displacement and separation from family members.^10 27^ Suicide attempts and suicidal ideation were linked to post-traumatic stress disorder (PTSD),^42 45 51 52^depression,^42 51^anxiety,^42^ sleep problems^28 51^ and substance use,^49 53^ prolonged grief disorder,^54^ and social fear and internet addiction.^53^ A family history of suicide^42^ or exposure to suicides in the family increased the risk for suicidal behaviours.^41 49^ Post-war stressful events further increased the risk.^49^ In Sweden, those who had migrated because of war showed higher suicide rates compared with non-war migrants.^37^ However, two studies reported lower suicide attempts or suicidal ideation rates among war-affected refugee children^55^ and immigrants compared with natives.^56^ Two studies reported family or social support as protective factors for suicide attempts and suicidal ideation.^28 40^

## Discussion

This scoping review provided an overview of studies examining suicides, suicide attempts and suicidal ideation among children and young people exposed to war or armed conflict. The review included 37 observational studies from 24 countries, covering a period of 100 years (1921–2025). Included studies spanned six continents, from Latin America (n=5, Colombia only) to Europe (n=10). There is an under-representation of research from most conflict-affected regions, and studies from Latin America were only represented by studies from Colombia. We found some evidence indicating a decrease in suicide rates during war, but no distinct trend was discernible in the post-war period. The prevalence estimates of suicide attempts and suicidal ideation varied widely and without any uniformity in definitions used, making comparisons across groups challenging.

### Suicide rates during war

There was some evidence of a decrease in suicide rates among children and young people during war, which is consistent with reviews on adults.^57 58^ The decreased suicide rates among 15–24-year-olds were observed during wartime in Sri Lanka,^36^ Bosnia and Herzegovina,^34^ Japan^39^ and England during World War II^38^; however, suicide rates in 15–24-year-old men rose during World War II in Scotland^31^ and the Homeland War in Croatia.^33^ One suggested reason for the reduced number of suicides during war has been linked to the economy and reduced unemployment.^58^ Another possible explanation may relate to increased connectedness with one’s own community during times of hardship, which may emerge in certain geopolitical contexts. Echoing the 3ST,^14^ connectedness to other people or meaningful goals may act as a protective factor against adverse psychological reactions and suicidality. This connectedness can be examined through social identity theory,^59^ which posits that individuals maintain a positive self-concept in part through affiliation with social groups (ie, in-groups), which are based for example on nationality, religion, ethnicity or political attitudes. In times of threats towards the in-group, the importance of this group identity and group loyalty can increase,^60^ and the increased social identification again may have a positive association with psychological well-being for some individuals.^61^

### Suicide rates post-war

Inconsistent findings were found in the suicide rates after the war. Increased suicide rates were observed in post-war Bosnia and Herzegovina^34^ and post-World War II England and Wales.^38^ One study reported a decline in suicide rates in 10–19-year-old adolescents after the Bosnian War,^35^ while the rates remained quite stable, followed by a high spike in 15–24-year-old youth in the USA after World War II.^32^ Another study reported high suicide rates early post-World War II in the UK, followed by a drop and later rise of suicide rates in men only.^38^ It is noteworthy that these results may reflect historico-cultural changes in health-seeking behaviour, in the social perception of psychological distress and traumatic events,^8^ as well as the concept of mental health itself.

A high number of weapon-related suicides was observed during the Homeland War in Croatia and the early postwar period.^33^ This is in line with the 3ST^14^ and with the evidence indicating that firearm availability is associated with a higher risk for suicide,^62^ particularly among young individuals.^63^

Inconsistent findings in the suicide rates after war were in line with previous studies among adults.^64^ In the aftermath of armed conflict, state institutions may undergo restructuring, or their functions may be affected by instability and resource constraints, thus affecting the availability of records and services. The decrease in youth suicide rates after the Bosnian War was reported to be due to a modification in suicide registration, leading to under-reporting of suicides.^35^ In terms of services, war inflicts detrimental effects on infrastructure and social and human resources, which can lead to significant gaps in support systems. Additionally, in modern wars, the neutrality principle of the healthcare sector^65^ has been increasingly violated, and healthcare services are becoming strategic targets of attacks.^9 66^ As a result, public mental and somatic health needs as well as social needs, already exacerbated by war, may be inadequately addressed and worsened over time. Stigmatisation and criminalisation of suicidal behaviour may also contribute to suicidality being under-reported and prevent help-seeking. War-related trauma, mental disorders, unemployment, hopelessness and uncertainty of the future^67^ are all likely to contribute to increased suicide rates. As per the 3ST,^14^ these represent mental health and experiential factors that contribute to psychological or emotional pain and, without hope for improvement, eventually decrease the individual’s will to live.

As for suicide attempts and suicidal ideation, the prevalence rates varied widely. This could be due to heterogeneity in definitions, populations, measurement tools, age of included populations and time when data were collected after armed conflicts. High prevalence rates of suicidal behaviours were reported among girls; however, suicides were reported more in boys. The findings were consistent with the ‘gender paradox of suicidal behaviour’,^68 69^ which refers to the phenomenon that men die more often by suicide than women, although women attempt suicide more frequently.^34 49 53^

In this review, refugees did not have an increased risk for suicides, suicide attempts or suicidal ideations.^37 55 70^ The survival drive for refugees could be high, and access to routine mental health services in the host country might result in lower rates of suicidal behaviour.^71^ Refugees may also not constitute a representative sample of the war-affected population in the country of origin; for example, individuals with severe mental disorders may not be able to take part in the often long and dangerous journey to seek asylum. Results regarding refugees should hence be interpreted with caution. The heterogeneity of refugee studies, conducted either in refugee camps, close to war zones or resettled in developed or developing countries, the attitude of the host country may influence the development of mental health outcomes.^72^ Additionally, the length of stay in the host country has been suggested as a potential mediator, with refugees having a lower risk initially, but after living in the new country for a longer period, the suicide risk has been reported to converge to the level of the host population.^37^ There is a need for further research in this population to understand the mechanisms contributing to risk and to explore protective factors from different countries.

### War and youth mental health

War, by its very nature, exposes the affected population to devastating adversities and potentially traumatic events. As one such example, several conflicts included in this review were characterised by instances of one-sided violence towards civilian groups. Such incidents are potentially highly traumatic, particularly if the perpetrators represent the in-group (eg, same nationality) and someone trusted, whereby the violation may be perceived as even more deleterious.^73^ Exposure to war atrocities has been associated with mental health problems (such as PTSD, depression and anxiety), which are antecedents to suicidal behaviours.^41 74 75^ Distressing media content of war,^76^ experiencing war events, stressful events after war and subsequent forced displacements further perpetuate the risk of mental health problems and may increase suicidal behaviours as a way to deal with symptoms.^74^ Children born to parents with mental disorders were at increased risk for mental disorders,^77 78^ and exposure to suicide, suicide attempts or suicidal ideation among families or friends was also linked with suicide attempts.^49 79^ Community, domestic^44 48^ or psychological abuse^53^ was associated with higher suicidal behaviour within the context of war. Such witnessed and direct exposure to violence might cumulate the effect of war-related trauma in children and young people, further undermining their perceived sense of safety in the world^80^ and thus making them more vulnerable to mental disorders and suicide. On the other hand, our review showed that family and social support^28 40^ were important protective factors of suicidal behaviours, reflecting earlier literature showing that the level and quality of family support systems, as well as the child or adolescent’s developmental stage and resilience factors, play a role in the psychological and emotional reactions following war trauma exposure.^81^

When managing adverse emotions, a harmful strategy may involve substance use.^82^ In the present review, substance use was found to be associated with a higher risk for suicidal ideation.^49 53 55^ Difficulties or maladaptation in emotion regulation, which are associated with PTSD in children and adolescents^83^ and anxiety in youth,^84^ are also associated with increased suicidal ideation and attempts in adolescents.^85^ This association may be further strengthened by guilt or shame, which exert a powerful impact on the individual and are linked to both suicidal ideation^86^ and PTSD, especially among those who have experienced war-related trauma.^87^ Guilt and shame can emerge from transgressions of moral or ethical norms^88^ or as survivor’s guilt,^89^ phenomena likely to appear within the context of war.

The mental health of children and young people in armed conflict regions is a dynamic process that requires an integrative ecological approach involving the family, school and community within their political, economic and cultural contexts.^90^ Children’s and adolescents’ adverse reactions linked to wars should not be analysed merely through a medicalised lens, but understanding the extent of war-related psychological suffering and the factors contributing to it is of importance when designing preventive, protective and curative measures. Regrettably, the principle of protecting civilians from the horrors of war is often violated; nevertheless, researchers have an important role in developing empirically supported post-conflict recovery measures. Future research should identify mediating and moderating factors related to suicidality, including cognitive, social and emotional factors, and investigate their interaction with broader mental health outcomes.

Our review included studies across 24 countries and regions/continents worldwide. To our knowledge, this is the first scoping review on suicides, suicide attempts and suicidal ideation among children and young people exposed to war or armed conflict, as previous studies were mainly focused on PTSD, depression and anxiety. However, our review has several limitations. First, due to available resources within our research team, we only included English-language articles. War and armed conflicts occur across the world; therefore, it is possible that some publications may only be available in local languages. Second, given the difficulty of assessing suicidal behaviours after war or armed conflicts, most studies used convenience sampling and thus might lack representativeness. Third, the assessment tools were heterogeneous, limiting our ability to draw firm conclusions across studies and limiting generalisability. Fourth, there is under-representation of some major conflict zones, namely Africa or the Middle East, where larger populations of children and young adults are affected by ongoing wars.

## Conclusions

An unprecedented number of children are exposed to war or armed conflict each year; however, methodologically rigorous research on mental health consequences is limited. Most studies were from Western countries. A future, larger multilingual review of the studies is required to provide a comprehensive understanding of the empirical evidence on the war on the mental health of children and young people. Family and social support were identified as protective factors. Conversely, risk factors encompassed exposure to war, traumatic events, stressful events after war, substance abuse and exposure to suicide or suicidal behaviours. Future research should identify mediating and moderating factors related to suicidality, including cognitive, social and emotional factors and their links to mental health.

## Supplementary material

10.1136/bmjopen-2025-103126 online supplemental file 1

## Data Availability

All data relevant to the study are included in the article or uploaded as supplementary information.

## References

[1] Khorram-Manesh A, Burkle FM, Goniewicz K (2021). Estimating the Number of Civilian Casualties in Modern Armed Conflicts-A Systematic Review. Front Public Health.

[2] Honwana A (2008). Children’s Involvement in War: Historical and Social Contexts. *hcy*.

[3] UN (2023). Children and armed conflict.

[4] Watson Institute for International & Public Affairs (2024). Civilians Killed & Wounded | Costs of War.

[5] Hazer L, Gredebäck G (2023). The effects of war, displacement, and trauma on child development. *Humanit Soc Sci Commun*.

[6] Bürgin D, Anagnostopoulos D, Anagnostopoulos D (2022). Impact of war and forced displacement on children’s mental health—multilevel, needs-oriented, and trauma-informed approaches. Eur Child Adolesc Psychiatry.

[7] Murthy RS, Lakshminarayana R (2006). Mental health consequences of war: a brief review of research findings. World Psychiatry.

[8] Summerfield D (1996). The psychological legacy of war and atrocity: the question of long-term and transgenerational effects and the need for a broad view. J Nerv Ment Dis.

[9] Pedersen D (2002). Political violence, ethnic conflict, and contemporary wars: broad implications for health and social well-being. Soc Sci Med.

[10] Sourander A, Silwal S, Osokina O (2024). Suicidality and Self-Harm Behavior of Adolescents During the Early Phase of the War in Ukraine. J Am Acad Child Adolesc Psychiatry.

[11] WHO (2025). Mental health of adolescents.

[12] Andover MS, Morris BW, Wren A (2012). The co-occurrence of non-suicidal self-injury and attempted suicide among adolescents: distinguishing risk factors and psychosocial correlates. Child Adolesc Psychiatry Ment Health.

[13] Kirshenbaum JS, Pagliaccio D, Bitran A (2024). Why do adolescents attempt suicide? Insights from leading ideation-to-action suicide theories: a systematic review. Transl Psychiatry.

[14] Klonsky ED, May AM (2015). The Three-Step Theory (3ST): A New Theory of Suicide Rooted in the “Ideation-to-Action” Framework. Int J Cogn Ther.

[15] UCDP (2025). UCDP: Sharp increase in conflicts and wars – Uppsala University.

[16] Sechser TS, Narang N, Talmadge C (2019). Emerging technologies and strategic stability in peacetime, crisis, and war. J Strateg Stud.

[17] Meddings DR (2001). Civilians and war: a review and historical overview of the involvement of non-combatant populations in conflict situations. Med Confl Surviv.

[18] UNICEF (2024). Child Displacement and Refugees.

[19] Bendavid E, Boerma T, Akseer N (2021). The effects of armed conflict on the health of women and children. Lancet.

[20] Kadir A, Shenoda S, Goldhagen J (2019). Effects of armed conflict on child health and development: A systematic review. PLoS One.

[21] Kien C, Sommer I, Faustmann A (2019). Prevalence of mental disorders in young refugees and asylum seekers in European Countries: a systematic review. Eur Child Adolesc Psychiatry.

[22] WHO (2003). Adolescent friendly health services: an agenda for change. https://apps.who.int/iris/handle/10665/67923.

[23] Cunningham H (1998). Histories of Childhood. Am Hist Rev.

[24] Page A, Morrell S, Taylor R (2002). Suicide and political regime in New South Wales and Australia during the 20th century. J Epidemiol Community Health.

[25] Sawyer SM, Azzopardi PS, Wickremarathne D (2018). The age of adolescence. The Lancet Child & Adolescent Health.

[26] NIH (2025). Suicide - National Institute of Mental Health (NIMH).

[27] Harrison R, Jones B, Gardner P (2021). Quality assessment with diverse studies (QuADS): an appraisal tool for methodological and reporting quality in systematic reviews of mixed- or multi-method studies. BMC Health Serv Res.

[28] Itani T, Jacobsen KH, Kraemer A (2017). Suicidal ideation and planning among Palestinian middle school students living in Gaza Strip, West Bank, and United Nations Relief and Works Agency (UNRWA) camps. Int J Pediatr Adolesc Med.

[29] UCDP (2023). UCDP - Uppsala Conflict Data Program.

[30] Pettersson T (2024). UDCP One-sided Violence Codebook Version 24.1.

[31] Henderson R, Stark C, Humphry RW (2006). Changes in Scottish suicide rates during the Second World War. BMC Public Health.

[32] McCall PL (1991). Adolescent and Elderly White Male Suicide Trends: Evidence of Changing Well-being?. J Gerontol.

[33] Mujkic A, Peek-Asa C, Young T (2008). Effect of war on weapon-related deaths in Croatian children and youth. Arch Pediatr Adolesc Med.

[34] Santic Z, Ostojic L, Hrabac B (2010). Suicide frequency in West-Herzegovina Canton for the period 1984-2008. Med Arh.

[35] Fajkic A, Lepara O, Voracek M (2010). Child and adolescent suicides in Bosnia and Herzegovina before and after the war (1992-1995). Crisis.

[36] Somasundaram DJ, Rajadurai S (1995). War and suicide in northern Sri Lanka. Acta Psychiatr Scand.

[37] Thordardottir EB, Yin L, Hauksdottir A (2020). Mortality and major disease risk among migrants of the 1991-2001 Balkan wars to Sweden: A register-based cohort study. PLoS Med.

[38] Murphy E, Lindesay J, Grundy E (1986). 60 years of suicide in England and Wales. A cohort study. Arch Gen Psychiatry.

[39] Kageyama T, Naka K (1996). Longitudinal change in youth suicide mortality in Okinawa after World War II: a comparative study with mainland Japan. Psychiatry Clin Neurosci.

[40] Jan M, Rather Y, Majeed N (2017). Psychosocial risk factors and clinical profile associated with attempted suicide in young adult and adolescent patients in conflict zone-Kashmir. Ann Trop Med Public Health.

[41] Jegannathan B, Kullgren G (2011). Gender differences in suicidal expressions and their determinants among young people in Cambodia, a post-conflict country. BMC Psychiatry.

[42] Kassa MA, Srahbzu M, Nakie G (2022). Suicidal ideation and attempts among high school students of war- affected area at Woldia town, Northeast, Ethiopia, 2022. BMC Psychiatry.

[43] Marroquín Rivera A, Rincón Rodríguez CJ, Padilla-Muñoz A (2020). Mental health in adolescents displaced by the armed conflict: findings from the Colombian national mental health survey. Child Adolesc Psychiatry Ment Health.

[44] Orrego S, Hincapié GMS, Restrepo D (2020). Mental disorders in the context of trauma and violence in a population study. Rev Colomb Psiquiatr (Engl Ed).

[45] Scharpf F, Masath FB, Mkinga G (2024). Prevalence of suicidality and associated factors of suicide risk in a representative community sample of families in three East African refugee camps. *Soc Psychiatry Psychiatr Epidemiol*.

[46] Olema DK, Catani C, Ertl V (2014). The hidden effects of child maltreatment in a war region: correlates of psychopathology in two generations living in Northern Uganda. J Trauma Stress.

[47] Ortega-Narváez A, Muñoz-Manquillo DM, Guzmán-Lopez CP (2024). Profiles of suicide attempted in children and adolescents. J Pediatr (Rio J).

[48] Moe CA, Villaveces A, Rivara FP (2022). Self-harming behavior in relation to exposure to inter-personal violence among youth and young adults in Colombia. Int J Inj Contr Saf Promot.

[49] Arënliu A (2014). Suicide Ideation and Behavior of Kosovar Adolescents: Effect of Negative Life Events, Reported Wellbeing, Happiness. Coping Mechanisms and Self-Esteem.

[50] Kinyanda E, Weiss HA, Mungherera M (2013). Prevalence and risk factors of attempted suicide in adult war-affected population of eastern Uganda. Crisis.

[51] Hamdan S, Hallaq E (2021). Prolonged exposure to violence: Psychiatric symptoms and suicide risk among college students in the Palestinian territory. *Psychol Trauma*.

[52] Wolde A, Dessalegn N (2022). Posttraumatic Stress Disorder, Suicidal Behavior, Substance Use, and Sexual Victimization Among Adolescent Girls Aged 10-19 Years Living Under Ethnic-Based Civil War in Ethiopia. Neuropsychiatr Dis Treat.

[53] Chahine M, Salameh P, Haddad C (2020). Suicidal ideation among Lebanese adolescents: scale validation, prevalence and correlates. BMC Psychiatry.

[54] Morina N, von Lersner U, Prigerson HG (2011). War and Bereavement: Consequences for Mental and Physical Distress. *PLoS ONE*.

[55] Betancourt TS, Newnham EA, Birman D (2017). Comparing Trauma Exposure, Mental Health Needs, and Service Utilization Across Clinical Samples of Refugee, Immigrant, and U.S.‐Origin Children. J Trauma Stress.

[56] Betancourt TS, Newnham EA, Layne CM (2012). Trauma History and Psychopathology in War‐Affected Refugee Children Referred for Trauma‐Related Mental Health Services in the United States. J Trauma Stress.

[57] Aida T (2020). Revisiting suicide rate during wartime: Evidence from the Sri Lankan civil war. PLoS ONE.

[58] Lester D, Wasserman D, Wasserman C (2009). Oxford textbook of suicidology and suicide prevention.

[59] Tajfel H, Turner JC (2004). Political psychology: key readings.

[60] Biruski DC, Ajdukovic D, Stanic AL (2014). When the world collapses: changed worldview and social reconstruction in a traumatized community. Eur J Psychotraumatol.

[61] Schmid K, Muldoon OT (2015). Perceived Threat, Social Identification, and Psychological Well‐Being: The Effects of Political Conflict Exposure. Polit Psychol.

[62] Westefeld JS, Gann LC, Lustgarten SD (2016). Relationships between firearm availability and suicide: The role of psychology. Professional Psychology: Research and Practice.

[63] Lowry NJ, Stanley IH, Mournet AM (2023). Firearms Access among Pediatric Patients at Risk for Suicide. Arch Suicide Res.

[64] Levav I, Klomek AB (2018). A review of epidemiologic studies on suicide before, during, and after the Holocaust. Psychiatry Res.

[65] International Committee of the Red Cross (1949). Geneva Conventions of 12 August 1949.

[66] Ghebreyesus TA (2024). Attacks on health are becoming the new reality; we must stop this becoming the norm.

[67] Bryan CJ, Hernandez AM, Allison S (2013). Combat Exposure and Suicide Risk in Two Samples of Military Personnel. J Clin Psychol.

[68] Barrigon ML, Cegla-Schvartzman F, Baca-Garcia E (2020). Behavioral neurobiology of suicide and self harm.

[69] Michaud L, Brovelli S, Bourquin C (2021). The gender paradox in suicide: some explanations and much uncertainty. Rev Med Suisse.

[70] Slodnjak V, Kos A, Yule W (2002). Depression and parasuicide in refugee and Slovenian adolescents. Crisis.

[71] Bakhiyi CL, Calati R, Guillaume S (2016). Do reasons for living protect against suicidal thoughts and behaviors? A systematic review of the literature. J Psychiatr Res.

[72] Blackmore R, Gray KM, Boyle JA (2020). Systematic Review and Meta-analysis: The Prevalence of Mental Illness in Child and Adolescent Refugees and Asylum Seekers. J Am Acad Child Adolesc Psychiatry.

[73] Goldsmith RE, Freyd JJ, DePrince AP (2012). Betrayal trauma: associations with psychological and physical symptoms in young adults. J Interpers Violence.

[74] Cepuch G, Kruszecka-Krówka A, Liber P (2023). Association between Suicidal Behaviors in Adolescence and Negative Emotions, the Level of Stress. Healthcare (Basel).

[75] Sami H, Hallaq E (2018). Nonsuicidal self-injury among adolescents and young adults with prolonged exposure to violence: The effect of post-traumatic stress symptoms. Psychiatry Res.

[76] Pfefferbaum B, Tucker P, Varma V (2020). Children’s Reactions to Media Coverage of War. Curr Psychiatry Rep.

[77] Joelsson P, Chudal R, Uotila J (2017). Parental psychopathology and offspring attention-deficit/hyperactivity disorder in a nationwide sample. J Psychiatr Res.

[78] Jokiranta E, Brown AS, Heinimaa M (2013). Parental psychiatric disorders and autism spectrum disorders. Psychiatry Res.

[79] O’Connor RC, Rasmussen S, Hawton K (2014). Adolescent self-harm: a school-based study in Northern Ireland. J Affect Disord.

[80] Collins KS (2001). Children’s Perceptions of Safety and Exposure to Violence. Int J Adolesc Youth.

[81] Pine DS, Costello J, Masten A (2005). Trauma, proximity, and developmental psychopathology: the effects of war and terrorism on children. Neuropsychopharmacology.

[82] Weiss NH, Kiefer R, Goncharenko S (2022). Emotion regulation and substance use: A meta-analysis. Drug Alcohol Depend.

[83] Villalta L, Smith P, Hickin N (2018). Emotion regulation difficulties in traumatized youth: a meta-analysis and conceptual review. Eur Child Adolesc Psychiatry.

[84] Schäfer JÖ, Naumann E, Holmes EA (2017). Emotion Regulation Strategies in Depressive and Anxiety Symptoms in Youth: A Meta-Analytic Review. J Youth Adolesc.

[85] Colmenero-Navarrete L, García-Sancho E, Salguero JM (2022). Relationship Between Emotion Regulation and Suicide Ideation and Attempt in Adults and Adolescents: A Systematic Review. Arch Suicide Res.

[86] Kealy D, Treeby MS, Rice SM (2021). Shame, guilt, and suicidal thoughts: The interaction matters. Br J Clin Psychol.

[87] Kip A, Diele J, Holling H (2022). The relationship of trauma-related guilt with PTSD symptoms in adult trauma survivors: a meta-analysis. Psychol Med.

[88] Tangney JP, Stuewig J, Mashek DJ (2007). Moral emotions and moral behavior. Annu Rev Psychol.

[89] Murray H, Pethania Y, Medin E (2021). Survivor Guilt: A Cognitive Approach. *Cogn Behav Therap*.

[90] Betancourt TS, Khan KT (2008). The mental health of children affected by armed conflict: Protective processes and pathways to resilience. Int Rev Psychiatry.

