# Supplementary material for: Suicides, suicide attempts and suicidal ideation among children and young people exposed to war: a scoping review
Source: BMJ Open. 2026 Jun 4;16(6):e103126. doi: 10.1136/bmjopen-2025-103126 (PMC13239371; doi:10.1136/bmjopen-2025-103126)
Supplement: online supplemental file 1 [file bmjopen-16-6-s001.docx]

**Supplemental Materials**

**Contents**

[**Supplemental Material 1. Search strategies** 2](#_Toc218680371)

[**Supplementary appendix Table 2. Attempts to contact author** 4](#_Toc218680372)

[**Supplemental Material 3. List of excluded studies** 5](#_Toc218680373)

[**Supplementary appendix Table 4: Summary of included studies** 13](#_Toc218680374)

[**Supplementary appendix Table 5: Summary of competed suicides, suicide attempts and suicidal ideation** 18](#_Toc218680375)

[**Supplementary appendix Table 6. Quality assessment of the studies included in the review** 26](#_Toc218680376)

# **Supplemental Material 1. Search strategies**

We searched Web of Science, PubMed, EMBASE and PsychINFO. The searches were conducted for studies published up to 17 March 2024. Below are the search strategies used in each database.

***Database: PubMed search strategy***

("Armed Conflicts"[Mesh] OR War[tw] OR wars*[tw] OR warfare* OR "armed conflict*" OR "complex emergenc*") AND ("Suicide"[Mesh] OR "suicidal ideation"[MeSH Terms] OR suicide* OR suicidal* OR "suicide attempt*" OR "suicidal ideation*" OR "suicidal thought*") AND ("child, preschool"[MeSH Terms] OR "Child"[MeSH Terms] OR "Adolescent"[MeSH Terms] OR "Infant"[MeSH Terms] OR Infant* OR infanc* OR Newborn* OR Baby* OR Babies* OR Child* OR Schoolchild* OR "School age*" OR Preschool* OR Kid OR kids OR Toddler* OR Adoles* OR Teen* OR Minors* OR Paediatric* OR Pediatric* OR "Nursery school*" OR Kindergar* OR "Primary school*" OR "Secondary school*" OR "Elementary school*" OR "High school*" OR Highschool* OR preteen* OR youth* OR young[tw] OR youngster* OR "unaccompanied minor*" OR "Refugees" [Mesh] OR refugee* OR "displaced person*" OR "asylum seeker*" OR "stateless person*" OR "war survivor*" OR "conflict survivor*" OR "war victim*" OR exile OR "uprooted person*" OR "Transients and Migrants"[Mesh] OR migrant*)

***Database: Web of Science search strategy***

((TS=(war OR conflict OR “complex emergenc*”)) AND TS=(suicide* OR “suicide attempt*” OR “suicidal ideation*” OR “suicidal thought*”)) AND TS=(Infant* OR infanc* OR Newborn* OR Baby OR Babies OR Child* OR Schoolchild* OR "School age*" OR Preschool* OR Kid OR kids OR Toddler* OR adoles* OR Teen* OR Minors* OR Paediatric* OR Pediatric* OR "Nursery school*" OR Kindergar* OR "Primary school*" OR "Secondary school*" OR "Elementary school*" OR "High school*" OR Highschool* OR “young adult*” OR “young people” OR “young person” OR preteen OR youth* OR youngster* OR student* OR “unaccompanied minor*” OR refugee* OR “displaced person*” OR “asylum seeker*” OR “stateless person*” OR “war survivor*” OR “conflict survivor*” OR “war victim*” OR exile OR “uprooted person*” OR migrant)

***Database:* *Embase search strategy***

#4 #1 AND #2 AND #3 AND [embase]/lim

#3 infant*:ti,ab OR infanc*:ti,ab OR newborn*:ti,ab OR baby:ti,ab OR babies:ti,ab OR child*:ti,ab OR schoolchild*:ti,ab OR 'school age*':ti,ab OR preschool*:ti,ab OR kid:ti,ab OR kids:ti,ab OR toddler*:ti,ab OR adoles*:ti,ab OR teen*:ti,ab OR minors*:ti,ab OR paediatric*:ti,ab OR pediatric*:ti,ab OR 'nursery school*':ti,ab OR kindergar*:ti,ab OR 'primary school*':ti,ab OR 'secondary school*':ti,ab OR 'elementary school*':ti,ab OR 'high school*':ti,ab OR highschool*:ti,ab OR 'young adult*':ti,ab OR 'young people':ti,ab OR 'young person':ti,ab OR preteen:ti,ab OR youth*:ti,ab OR youngster*:ti,ab OR student*:ti,ab OR 'unaccompanied minor*':ti,ab OR refugee*:ti,ab OR 'displaced person*':ti,ab OR 'asylum seeker*':ti,ab OR 'stateless person*':ti,ab OR 'war survivor*':ti,ab OR 'conflict survivor*':ti,ab OR 'war victim*':ti,ab OR exile:ti,ab OR 'uprooted person*':ti,ab OR migrant:ti,ab

#2 suicide*:ti,ab OR 'suicide attempt*':ti,ab OR 'suicidal ideation*':ti,ab OR 'suicidal thought*':ti,ab

#1 war:ti,ab OR conflict:ti,ab OR 'complex emergenc*':ti,ab

***Database:* *PsycINFO via EBSCOHost search strategy***

S1 TI ( war OR conflict OR “complex emergenc*” ) OR AB ( war OR conflict OR “complex emergenc*” )

S2 TI ( suicide* OR “suicide attempt*” OR “suicidal ideation*” OR “suicidal thought*”) OR AB ( suicide* OR “suicide attempt*” OR “suicidal ideation*” OR “suicidal thought*”)

S3 TI ( Infant* OR infanc* OR Newborn* OR Baby OR Babies OR Child* OR Schoolchild* OR "School age*" OR Preschool* OR Kid OR kids OR Toddler* OR adoles* OR Teen* OR Minors* OR Paediatric* OR Pediatric* OR "Nursery school*" OR Kindergar* OR "Primary school*" OR "Secondary school*" OR "Elementary school*" OR "High school*" OR Highschool* OR “young adult*” OR “young people” OR “young person” OR preteen OR youth* OR youngster* OR student* OR “unaccompanied minor*” OR refugee* OR “displaced person*” OR “asylum seeker*” OR “stateless person*” OR “war survivor*” OR “conflict survivor*” OR “war victim*” OR exile OR “uprooted person*” OR ‘‘migrant’’ ) OR AB ( Infant* OR infanc* OR Newborn* OR Baby OR Babies OR Child* OR Schoolchild* OR "School age*" OR Preschool* OR Kid OR kids OR Toddler* OR adoles* OR Teen* OR Minors* OR Paediatric* OR Pediatric* OR "Nursery school*" OR Kindergar* OR "Primary school*" OR "Secondary school*" OR "Elementary school*" OR "High school*" OR Highschool* OR “young adult*” OR “young people” OR “young person” OR preteen OR youth* OR youngster* OR student* OR “unaccompanied minor*” OR refugee* OR “displaced person*” OR “asylum seeker*” OR “stateless person*” OR “war survivor*” OR “conflict survivor*” OR “war victim*” OR exile OR “uprooted person*” OR migrant )

S4 S1 AND S2 AND S3

# **Supplementary appendix Table 2. Attempts to contact author**

We contacted the author for additional information on the study. Reminder emails were sent a week after the first email.

| **Study** | **Contacted** | **Replied** | **Provided additional information** | **Notes** |
| --- | --- | --- | --- | --- |
| Betancourt et al. (2015) | Yes | Yes | Referred | The information was received from other organization. |

# **Supplemental Material 3. List of excluded studies**

**REFERENCES**

1. Acosta, D. S., Perez, G. A. C., Hincape, G. M. S., Vasquez, N. S. M., Zapata, C. S., Salazar, J. C. B., & de Galvis, Y. T. (2019). Mental Health of Adolescents and Young People Victims of Forced Displacement in Colombia. *REVISTA CES PSICOLOGIA*, *12*(3), 1-18. https://doi.org/10.21615/cesp.12.3.1
2. Alley, J. C. (1982). Life-threatening indicators among the Indochinese refugees. *Suicide Life Threat Behav*, *12*(1), 46-51. https://doi.org/10.1111/j.1943-278x.1982.tb01092.x
3. Amone-P'Olak, K., Lekhutlile, T. M., Meiser-Stedman, R., & Ovuga, E. (2014). Mediators of the relation between war experiences and suicidal ideation among former child soldiers in Northern Uganda: the WAYS study. *BMC Psychiatry*, *14*, 271. https://doi.org/10.1186/s12888-014-0271-2
4. An, J., Wang, T., Chen, B., Oleksiyenko, A., & Lin, C. (2025). Mental health of residents of Ukraine exposed to the russia-Ukraine conflict. *JAMA NETWORK OPEN*, *8*(2), e2459318-e2459318.
5. Anacona, C. A. R. P., J. R.; Méndez, J. H. M. (2024). Sex differences and variables associated with suicidal planning in Colombian adolescents. *INTERDISCIPLINARIA*, *41*(1).
6. Apter, A. (2010). Suicidal behaviour in adolescence. *The Canadian Journal of Psychiatry / La Revue canadienne de psychiatrie*, *55*(5), 271-273. https:// doi.org/10.1177/070674371005500501.
7. Araki, S., & Murata, K. (1987). Suicide in Japan: socioeconomic effects on its secular and seasonal trends. *Suicide Life Threat Behav*, *17*(1), 64-71. https://doi.org/10.1111/j.1943-278x.1987.tb00062.x
8. Ben Khelil, M., Gharbaoui, M., Farhani, F., Zaafrane, M., Harzallah, H., Allouche, M., Zhioua, M., & Hamdoun, M. (2016). Impact of the Tunisian Revolution on homicide and suicide rates in Tunisia. *Int J Public Health*, *61*(9), 995-1002. https://doi.org/10.1007/s00038-016-0834-8
9. Bhui, K., Abdi, A., Abdi, M., Pereira, S., Dualeh, M., Robertson, D., Sathyamoorthy, G., & Ismail, H. (2003). Traumatic events, migration characteristics and psychiatric symptoms among Somali refugees--preliminary communication. *Soc Psychiatry Psychiatr Epidemiol*, *38*(1), 35-43. https://doi.org/10.1007/s00127-003-0596-5
10. Boričević Maršanić, V., Aukst Margetić, B., Ožanić Bulić, S., Đuretić, I., Kniewald, H., Jukić, T., & Paradžik, L. (2015). Non-suicidal self-injury among psychiatric outpatient adolescent offspring of Croatian posttraumatic stress disorder male war veterans: Prevalence and psychosocial correlates. *Int J Soc Psychiatry*, *61*(3), 265-274. https://doi.org/10.1177/0020764014541248
11. Bosnar, A., Stemberga, V., Coklo, M., Koncar, G. Z., Definis-Gojanovic, M., Sendula-Jengic, V., & Katic, P. (2005). Suicide and the war in Croatia. *Forensic Sci Int*, *147 Suppl*, S13-16. https://doi.org/10.1016/j.forsciint.2004.09.086
12. Bosnar, A., Stemberga, V., Cuculic, D., Zamolo, G., Stifter, S., & Coklo, M. (2004). Suicide rate after the 1991-1995 War in Southwestern Croatia. *Arch Med Res*, *35*(4), 344-347. https://doi.org/10.1016/j.arcmed.2004.03.001
13. Bwesige, M. M., & Snider, L. (2021). Despair and Suicide-Related Behaviours in Palorinya Refugee Settlement, Moyo, Uganda. *INTERVENTION-INTERNATIONAL JOURNAL OF MENTAL HEALTH PSYCHOSOCIAL WORK AND COUNSELLING IN AREAS OF ARMED CONFLICT*, *19*(2), 224-232. https://doi.org/10.4103/INTV.INTV_12_21
14. Čatipović, V. (2001). Suicide of psychiatrically treated patients in the bjelovar-bilogora county during the 1989-1999 period. *Socijalna Psihijatrija*, *29*(2), 76-86. https://www.embase.com/search/results?subaction=viewrecord&id=L32612230&from=export
15. Catipović, V., Koić, E., & Sklebar, D. (2014). [Suicides in Bjelovar-Bilogora County in the war period, pre-war and post-war period, and the period of the economic expansion and recession]. *Lijec Vjesn*, *136*(11-12), 324-334.
16. Cebula, R. J., & Zelenskaya, T. V. (2006). Determinants of youth suicide - A friendly comment with suggestions. *AMERICAN JOURNAL OF ECONOMICS AND SOCIOLOGY*, *65*(4), 991-996. https://doi.org/10.1111/j.1536-7150.2006.00486.x
17. Choi, S. W., & Piazza, J. A. (2016). Internally Displaced Populations and Suicide Terrorism. *JOURNAL OF CONFLICT RESOLUTION*, *60*(6), 1008-1040. https://doi.org/10.1177/0022002714550086
18. Drevinja, F., Berisha, B., Serreqi, V., Statovci, S., & Haxhibeqiri, S. (2013). Suicides in kosovo in five year period 2008-2012, an overview for possible social motives. *European Psychiatry*, *28*. https:// doi:10.1016/S0924-9338(13)77160-8
19. Ebuenyi, I. D., Chikezie, U. E., & Nwoke, E. A. (2021). Psychosocial correlates of risky sexual behaviour amongst students in Niger Delta University, Bayelsa. *PAN AFRICAN MEDICAL JOURNAL*, *38*. https://doi.org/10.11604/pamj.2021.38.7.27312
20. Ertl, V., Pfeiffer, A., Schauer-Kaiser, E., Elbert, T., & Neuner, F. (2014). The challenge of living on: psychopathology and its mediating influence on the readjustment of former child soldiers. *PLoS One*, *9*(7), e102786. https://doi.org/10.1371/journal.pone.0102786
21. Falb, K. L., McCormick, M. C., Hemenway, D., Anfinson, K., & Silverman, J. G. (2013). Suicide ideation and victimization among refugee women along the Thai–Burma border. *Journal of Traumatic Stress*, *26*(5), 631-635. https://doi.org/10.1002/jts.21846
22. Fekih-Romdhane, F., Jebreen, K., Swaitti, T., Jebreen, M., Radwan, E., Kammoun-Rebai, W., Abu Samra, M. A., Abusamra, A., Obeid, S., & Hallit, S. (2024). The indirect role of perceived survival expectations in the association between perceived hope and suicidal thoughts among palestinians amid war in Gaza. *SCIENTIFIC REPORTS*, *14*(1), 32035.
23. Ferrari, S., Artoni, C., Marchi, M., Longo, F., Magarini, F., Reggianini, C., & Galeazzi, G. (2020). Self-harm and suicide attempts among migrants in jail: the role of trauma and substance abuse. *Journal of Psychosomatic Research*, *133*. https://doi.org/10.1016/j.jpsychores.2020.110011
24. Flaskerud, J. H., & Anh, N. T. (1988). Mental health needs of Vietnamese refugees. *Hospital & Community Psychiatry*, *39*(4), 435-437. https://search.ebscohost.com/login.aspx?direct=true&db=psyh&AN=1988-26666-001&site=ehost-live
25. Forrest, W., Edwards, B., & Daraganova, G. (2018). The intergenerational consequences of war: anxiety, depression, suicidality, and mental health among the children of war veterans. *Int J Epidemiol*, *47*(4), 1060-1067. https://doi.org/10.1093/ije/dyy040
26. Franić, T., Dodig, G., Kardum, G., Marčinko, D., Ujević, A., & Bilušić, M. (2011). Early adolescence and suicidal ideations in Croatia: sociodemographic, behavioral, and psychometric correlates. *Crisis*, *32*(6), 334-345. https://doi.org/10.1027/0227-5910/a000107
27. GBD 2015 Eastern Mediterranean Region Adolescent Health Collaborators. (2018). Adolescent health in the Eastern Mediterranean Region: findings from the global burden of disease 2015 study. *Int J Public Health*, *63*(Suppl 1), 79-96. https://doi.org/10.1007/s00038-017-1003-4
28. GBD 2015 Eastern Mediterranean Region Intentional Injuries Collaborators. (2018). Intentional injuries in the Eastern Mediterranean Region, 1990-2015: findings from the Global Burden of Disease 2015 study. *Int J Public Health*, *63*(Suppl 1), 39-46. https://doi.org/10.1007/s00038-017-1005-2
29. Getanda, E. M., Papadopoulos, C., & Evans, H. (2015). The mental health, quality of life and life satisfaction of internally displaced persons living in Nakuru County, Kenya. *BMC PUBLIC HEALTH*, *15*. https://doi.org/10.1186/s12889-015-2085-7
30. Gilgen, D., Gross, C. S., Maeusezahl, D., Frey, C., Tanner, M., Weiss, M. G., & Hatz, C. (2002). Impact of organized violence on illness experience of Turkish/Kurdish and Bosnian migrant patients in primary care. *JOURNAL OF TRAVEL MEDICINE*, *9*(5), 236-243. https://doi.org/10.2310/7060.2002.24206
31. Grgić, M., Knežević, M. Z., Mandić, N., Degmečić, D., & Koić, O. (2001). Parasuicide amongst children and adolescents treated at the division of children's and adolescent psychiatry - Osijek. *Socijalna Psihijatrija*, *29*(4), 199-205. https://www.embase.com/search/results?subaction=viewrecord&id=L34118023&from=export
32. Grube, M. (2004). Nonfacial suicidal acts in a group of psychiatric inpatients. Situation of Mediterranean immigrants. *NERVENARZT*, *75*(7), 681-687. https://doi.org/10.1007/s00115-003-1648-5
33. Grubisić-Ilić, M., Kozarić-Kovacić, D., Grubisić, F., & Kovacić, Z. (2002). Epidemiological study of suicide in the Republic of Croatia -- comparison of war and post-war periods and areas directly and indirectly affected by war. *Eur Psychiatry*, *17*(5), 259-264. https://doi.org/10.1016/s0924-9338(02)00679-x
34. Hess, R. F., Croasmun, A. C., Pittman, C., Baird, M. B., & Ross, R. Psychological Distress, Post-Traumatic Stress, and Suicidal Ideation Among Resettled Nepali-Speaking Bhutanese Refugees in the United States: Rates and Predictors. *JOURNAL OF TRANSCULTURAL NURSING*. https://doi.org/10.1177/10436596211070599
35. Hill, S. A., Pritchard, C., Laugharne, R., & Gunnell, D. (2005). Changing patterns of suicide in a poor, rural county over the 20th century: a comparison with national trends. *Soc Psychiatry Psychiatr Epidemiol*, *40*(8), 601-604. https://doi.org/10.1007/s00127-005-0933-y
36. Hodes, M. (2010). The mental health of detained asylum seeking children. *EUROPEAN CHILD & ADOLESCENT PSYCHIATRY*, *19*(7), 621-623. https://doi.org/10.1007/s00787-010-0093-9
37. Ingabire, C. M., & Richters, A. (2020). Suicidal Ideation and Behavior Among Congolese Refugees in Rwanda: Contributing Factors, Consequences, and Support Mechanisms in the Context of Culture. *Front Psychiatry*, *11*, 299. https://doi.org/10.3389/fpsyt.2020.00299
38. Jankovic, J., Bremner, S., Bogic, M., Lecic-Tosevski, D., Ajdukovic, D., Franciskovic, T., Galeazzi, G. M., Kucukalic, A., Morina, N., Popovski, M., Schützwohl, M., & Priebe, S. (2013). Trauma and suicidality in war affected communities. *Eur Psychiatry*, *28*(8), 514-520. https://doi.org/10.1016/j.eurpsy.2012.06.001
39. Johnson, K., Asher, J., Rosborough, S., Raja, A., Panjabi, R., Beadling, C., & Lawry, L. (2008). Association of combatant status and sexual violence with health and mental health outcomes in postconflict Liberia. *JAMA-JOURNAL OF THE AMERICAN MEDICAL ASSOCIATION*, *300*(6), 676-690. https://doi.org/10.1001/jama.300.6.676
40. Kageyama, J., & Ishii, T. (1997). Comparisons of pre- and postwar suicide and homicide rates in Japan - Aggression and social problem. *Acta Criminologiae et Medicinae Legalis Japonica*, *63*(4), 122-132. https://www.embase.com/search/results?subaction=viewrecord&id=L27368714&from=export
41. Kapamadzija, B. (1976). Suicide in adolescents. *Psihijatrija Danas*, *8*(3-4), 361-370. https://search.ebscohost.com/login.aspx?direct=true&db=psyh&AN=1982-01437-001&site=ehost-live
42. Karam, E. G., Salamoun, M. M., Mneimneh, Z. N., Fayyad, J. A., Karam, A. N., Hajjar, R., Dimassi, H., Nock, M. K., & Kessler, R. C. (2012). War and first onset of suicidality: the role of mental disorders. *Psychol Med*, *42*(10), 2109-2118. https://doi.org/10.1017/s0033291712000268
43. Kizza, D., Hjelmeland, H., Kinyanda, E., & Knizek, B. L. (2012). Alcohol and suicide in postconflict northern Uganda: a qualitative psychological autopsy study. *Crisis*, *33*(2), 95-105. https://doi.org/10.1027/0227-5910/a000119
44. Kizza, D., Knizek, B. L., Kinyanda, E., & Hjelmeland, H. (2012). Men in despair: a qualitative psychological autopsy study of suicide in northern Uganda. *Transcult Psychiatry*, *49*(5), 696-717. https://doi.org/10.1177/1363461512459490
45. Kizza, D., Loa Knizek, B., Kinyanda, E., & Hjelmeland, H. (2012). An escape from agony: a qualitative psychological autopsy study of women's suicide in a post-conflict northern Uganda. *Int J Qual Stud Health Well-being*, *7*, 1-13. https://doi.org/10.3402/qhw.v7i0.18463
46. Kocijan-Hercigonja, D., & Remeta, D. (1996). Family conflicts as sources of mental health disturbances in infant and adolescent victims of war. *Croatian Medical Journal*, *37*(2), 105-110. https://www.embase.com/search/results?subaction=viewrecord&id=L26166151&from=export
47. Kozaric-Kovacic, D., Grubisic-Ilic, M., Grubisic, F., & Kovacic, Z. (2002). Epidemiological indicators of suicides in the Republic of Croatia. *DRUSTVENA ISTRAZIVANJA*, *11*(1), 155-170.
48. Lama, S., François, K., Marwan, Z., & Sami, R. (2016). Impact of the Syrian Crisis on the Hospitalization of Syrians in a Psychiatric Setting. *Community Ment Health J*, *52*(1), 84-93. https://doi.org/10.1007/s10597-015-9891-3
49. LeBouthillier, D. M., McMillan, K. A., Thibodeau, M. A., & Asmundson, G. J. (2015). Types and Number of Traumas Associated With Suicidal Ideation and Suicide Attempts in PTSD: Findings From a U.S. Nationally Representative Sample. *J Trauma Stress*, *28*(3), 183-190. https://doi.org/10.1002/jts.22010
50. Levi-Belz, Y., Amsalem, D., Groweiss, Y., Blank, C., Shachar-Lavie, I., & Neria, Y. (2025). A Year in the Shadow of Terror: Longitudinal Effects of the October 7, 2023, Terrorist Attack on PTSD, Depression, Anxiety, and Suicidal Ideation Across Distinct Exposure Groups. *The Journal of Clinical Psychiatry*, *86*(4), 25m15970-15925m15970.
51. Levi-Belz, Y., Blank, C., Groweiss, Y., & Neria, Y. (2024). The impact of PTSD symptoms on suicide ideation in time of terror and war: A nationwide prospective study on the moderating role of loneliness. *PSYCHIATRY RESEARCH*, *338*, 115996.
52. Louks, J. L., Otis, G. D., & Smith, J. R. (1999). Young female violent death trends in the general population during the Vietnam era. *Suicide Life Threat Behav*, *29*(3), 213-226. https://onlinelibrary.wiley.com/doi/abs/10.1111/j.1943-278X.1999.tb00298.x?sid=nlm%3Apubmed
53. Louks, J. L., Otis, G. D., Smith, J. R., Hayne, C. H., & Trent, H. E. (1999). Young male violent death trends in the general population during the Vietnam era. *Suicide Life Threat Behav*, *29*(3), 201-212. https://onlinelibrary.wiley.com/doi/abs/10.1111/j.1943-278X.1999.tb00297.x?sid=nlm%3Apubmed
54. Maalouf, F. (2025). 89.3 Psychopathology in Children and Adolescents in Lebanon Study: Insights and Cultural Considerations. *Journal of the American Academy of Child & Adolescent Psychiatry*, *64*(10), S125.
55. Marchi, M., Artoni, C., Longo, F., Magarini, F. M., Aprile, G., Reggianini, C., Florio, D., De Fazio, G. L., Galeazzi, G. M., & Ferrari, S. (2022). The impact of trauma, substance abuse, and psychiatric illness on suicidal and self-harm behaviours in a cohort of migrant detainees: An observational, prospective study. *Int J Soc Psychiatry*, *68*(3), 514-524. https://doi.org/10.1177/0020764020979007
56. Marković, H., & Marković, A. (1997). Parasuicid na dubrovačkom području = Parasuicide in the Dubrovnik area. *Socijalna Psihijatrija*, *25*(1-2), 32-37. https://search.ebscohost.com/login.aspx?direct=true&db=psyh&AN=1998-00418-004&site=ehost-live
57. Marzouk, H. A. (2021). International Organization for Migration Iraq Mental and Psychosocial Support Programme Suicide Prevention Activities. *INTERVENTION-INTERNATIONAL JOURNAL OF MENTAL HEALTH PSYCHOSOCIAL WORK AND COUNSELLING IN AREAS OF ARMED CONFLICT*, *19*(2), 255-260. https://doi.org/10.4103/INTV.INTV_6_21
58. McLafferty, M., Armour, C., O'Neill, S., Murphy, S., Ferry, F., & Bunting, B. (2016). Suicidality and profiles of childhood adversities, conflict related trauma and psychopathology in the Northern Ireland population. *Journal of Affective Disorders*, *200*, 97-102. https://doi.org/10.1016/j.jad.2016.04.031
59. Morina, N., & Emmelkamp, P. M. G. (2012). Mental health outcomes of widowed and married mothers after war. *BRITISH JOURNAL OF PSYCHIATRY*, *200*(2), 158-159. https://doi.org/10.1192/bjp.bp.111.093609
60. Mugisha, J., Muyinda, H., Kagee, A., Wandiembe, P., Mpugu, S. K., Vancampfort, D., & Kinyanda, E. (2016). Prevalence of suicidal ideation and attempt: associations with psychiatric disorders and HIV/AIDS in post-conflict Northern Uganda. *Afr Health Sci*, *16*(4), 1027-1035. https://doi.org/10.4314/ahs.v16i4.20
61. Mugisha, J., Muyinda, H., Malamba, S., & Kinyanda, E. (2015). Major depressive disorder seven years after the conflict in northern Uganda: burden, risk factors and impact on outcomes (The Wayo-Nero Study). *BMC Psychiatry*, *15*, 48. https://doi.org/10.1186/s12888-015-0423-z
62. Najem, Y., Malaeb, D., Sakr, F., Dabbous, M., Fekih-Romdhane, F., Hallit, S., & Obeid, S. (2025). Mediating effect of intolerance of uncertainty between fear of war and mental health in adults during the Israel-Palestine war of 2023. *DISCOVER MENTAL HEALTH*, *5*(1), 19.
63. O'Neill, S., & O'Connor, R. C. (2020). Suicide in Northern Ireland: epidemiology, risk factors, and prevention. *The Lancet Psychiatry*, *7*(6), 538-546. https://doi.org/10.1016/S2215-0366(19)30525-5
64. O'Neill, S., & Rooney, N. (2018). Mental health in Northern Ireland: An urgent situation. *The Lancet Psychiatry*, *5*(12), 965-966. https://doi.org/10.1016/S2215-0366(18)30392-4
65. O'Neill, S., & Rooney, N. (2019). 'Mental health in Northern Ireland: An urgent situation': Correction. *The Lancet Psychiatry*, *6*(1), e2-e2. https://doi.org/10.1016/S2215-0366(18)30489-9
66. Oppong Asante, K., Quarshie, E. N. B., & Onyeaka, H. K. (2021). Epidemiology of suicidal behaviours amongst school-going adolescents in post-conflict Sierra Leone. *Journal of Affective Disorders*, *295*, 989-996. https://doi.org/10.1016/j.jad.2021.08.147
67. Ostaszewski, K., Markiewicz, M., Klimanska, M., Haletska, I., Herasym, H., Klymanska, L., Mirchuk, I., Savka, V., & Zelena, O. (2025). Prevalence of and factors in suicidal thoughts in 14-15-year-olds. A Polish-Ukrainian study [journal article]. *Advances in Psychiatry and Neurology/Postępy Psychiatrii i Neurologii*, *34*(2), 75-86. https://doi.org/10.5114/ppn.2025.151776
68. Ovuga, E., Oyok, T. O., & Moro, E. B. (2008). Post traumatic stress disorder among former child soldiers attending a rehabilitative service and primary school education in northern Uganda. *Afr Health Sci*, *8*(3), 136-141. https://www.ncbi.nlm.nih.gov/pmc/articles/PMC2583264/pdf/AFHS0803-0136.pdf
69. Peltonen, K., Hakala, V., Laajasalo, T., Stenvall, E., Mielityinen, L., & Ellonen, N. (2025). The Expressed Worries of Ukrainian Adolescents: A Quantitative Analysis of Chat Conversations During Active War. *YOUNG*, *33*(2), 132-148.
70. Rahman, A., & Hafeez, A. (2003). Suicidal feelings run high among mothers in refugee camps: a cross-sectional survey. *ACTA PSYCHIATRICA SCANDINAVICA*, *108*(5), 392-393. https://doi.org/10.1034/j.1600-0447.2003.00220.x
71. Reza, A., Mercy, J. A., & Krug, E. (2001). Epidemiology of violent deaths in the world. *Inj Prev*, *7*(2), 104-111. https://doi.org/10.1136/ip.7.2.104
72. Rozynek, D., Śmierciak, N., Wojszel, B., Rodak, W., Bagieńska, M., Valynets-Cyganik, A., Slabucho, V., Talybov, S., Wojtasik-Bakalarz, K., & Szwajca, M. (2025). Ukrainian refugee crisis center at the University Hospital’s Psychiatric Clinic for Adults, Children and Adolescents in Krakow. *Advances in Psychiatry and Neurology/Postępy Psychiatrii i Neurologii*, *34*(1), 11-18.
73. Rubanzana, W., Hedt-Gauthier, B. L., Ntaganira, J., & Freeman, M. D. (2015). Exposure to genocide and risk of suicide in Rwanda: a population-based case-control study. *J Epidemiol Community Health*, *69*(2), 117-122. https://doi.org/10.1136/jech-2014-204307
74. Schlarb, A. A., Holdmann, L., & Bünnemann, M. (2020). Sleep disturbances and mental disorders in help-seeking unaccompanied refugee minors. *Journal of Sleep Research*, *29*(SUPPL 1). https://doi.org/10.1111/jsr.13181
75. Sumner, A. E., Whittall, J., Rodrigo, A., Da Silva, T., & Ravindran, A. V. (2017). Socioeconomic, psychosocial, and healthcare-access contributors to poisoning and suicide in sri lanka: An ecological survey. *Annals of Global Health*, *83*(1), 181-182. https://www.embase.com/search/results?subaction=viewrecord&id=L620061194&from=export
76. Surault, P. (1995). CHANGING PATTERNS IN SUICIDE IN FRANCE. *POPULATION*, *50*(4-5), 983-1012. https://doi.org/10.2307/1534312
77. Tabur, S., Tufan, A. E., Çeri, V., & Semerci, B. (2019). Syrian Civil War’s effects on Turkish school children: Prevalence and predictors of psychopathology. *Psychiatry and Clinical Psychopharmacology*, *29*(4), 811-816. https://doi.org/10.1080/24750573.2019.1661758
78. Tamayo Martínez, N., Rincón Rodríguez, C. J., de Santacruz, C., Bautista Bautista, N., Collazos, J., & Gómez-Restrepo, C. (2016). [Mental Problems, Mood and Anxiety Disorders in The Population Displaced by Violence in Colombia; Results of The National Mental Health Survey 2015]. *Rev Colomb Psiquiatr*, *45 Suppl 1*, 113-118. https://doi.org/10.1016/j.rcp.2016.09.004
79. Tomlinson, M. (2007). Suicide and young people: The case of Northern Ireland. *Child Care in Practice*, *13*(4), 435-443. https://doi.org/10.1080/13575270701504802 (Going from strength to strength-promoting children's mental health)
80. Tomlinson, M. W. (2012). War, peace and suicide: The case of Northern Ireland. *INTERNATIONAL SOCIOLOGY*, *27*(4), 464-482. https://doi.org/10.1177/0268580912443579
81. Trujillo, S., Giraldo, L. S., Lopez, J. D., Acosta, A., & Trujillo, N. (2021). Mental health outcomes in communities exposed to Armed Conflict Experiences. *BMC PSYCHOLOGY*, *9*(1). https://doi.org/10.1186/s40359-021-00626-2
82. Uwakwe, R., Oladeji, B. D., & Gureje, O. (2012). Traumatic events and suicidal behaviour in the Nigerian Survey of Mental Health and Well-Being. *Acta Psychiatr Scand*, *126*(6), 458-466. https://doi.org/10.1111/j.1600-0447.2012.01852.x
83. Veronese, G., Diab, M., Abu Jamei, Y., Saleh, S., & Kagee, A. (2021). Risk and protection of suicidal behavior among Palestinian University Students in the Gaza Strip: An exploratory study in a context of military violence. *INTERNATIONAL JOURNAL OF MENTAL HEALTH*, *50*(4), 293-310. https://doi.org/10.1080/00207411.2021.1911381
84. Wen, C.-P. (1974). Secular suicidal trend in postwar Japan and Taiwan: An examination of hypotheses. *International Journal of Social Psychiatry*, *20*(1-2), 8-17. https://doi.org/10.1177/002076407402000102
85. Wenzel, T., Rushiti, F., Aghani, F., Diaconu, G., Maxhuni, B., & Zitterl, W. (2009). Suicidal ideation, post-traumatic stress and suicide statistics in Kosovo. An analysis five years after the war. Suicidal ideation in Kosovo. *Torture*, *19*(3), 238-247.
86. Zhjeqi, V., Ramadani, N., Gashi, S., Mucaj, S., Berisha, M., Neziri, L., Krasniqi, S., & Shahini, M. (2010). Suicide prevalence in Kosova for the period 2007-2008. *Med Arh*, *64*(1), 44-47.

# **Supplementary appendix Table 4: Summary of included studies**

| **Author (Year)** | **Study design** | **Study period, Type of conflict (UCDP)** | **Population, Sample size, Age, Informants** | **Data source** |
| --- | --- | --- | --- | --- |
| **Africa** | | | | |
| Scharpf et al. (2024) | Cross-sectional | 2018, Burundian conflict  Intrastate; non-state, one-sided violence | Refugees, N=230, 7-15 years, interview | Refugee camps |
| Kassa et al. (2023) | Cross-sectional | 2022, Ethiopia, Woldia town  State-based; one-sided violence | High school students, N=668, 14-25 years, self-report | School survey |
| Wolde & Dessalegn (2022) | Cross-sectional | 2021, Ethiopia, civil war  State-based; one-sided violence | Girls from three towns, NA, M 14.3 (SD=2.8), interview | Community |
| Olema et al. (2014) | Cross-sectional | 2006, Civil war between Ugandan Government and Kony's LRA Rebel group (1986-2006)  Intrastate; non-state based; one-sided violence | Abducted and non-abducted children and parents, N=300 (n=50 abducted children, n=50 non-abducted children, n=200 parents), 12-17 years, interviews with children and parents | Interview in IDP camps |
| Okello et al. (2013) | Cross-sectional | 2010, Civil war between Ugandan Government and Kony's LRA Rebel group (1986-2006)  Intrastate; non-state based; one-sided violence | School-going adolescents, N=551, 13-21 years, self-report | Schools survey |
| Kinyanda et al. (2013) | Cross-sectional | N/A, Civil war against Lord's Resistance Army (LRA) rebel group (relative peace since 2004)  Intrastate; non-state based; one-sided violence | Subgroup of adolescents in two districts, N=445, 15-24 years, interviews with participants | Survey in four sub-counties in the two districts of Amuria and Katakwi in war-affected Eastern Uganda |
| Okello, Onnen & Musisi. (2007) | Cross-sectional | 2004, Civil war between Ugandan Government and Kony's LRA Rebel group (1986-2006)  Intrastate; non-state based; one-sided violence | Formerly abducted and non-abducted children, N=153 (n=82 abducted and n=71 non-abducted children), 11-19 years, interviewed children | Trauma reception center survey, college surveys |
| **Europe** | | | | |
| Sourander et al. (2024) | Cross-sectional | 2014 Russian invasion of Ukraine  Interstate; state-based, non-state based | Adolescents from war-affected and non-war region, N=2752 (n=1449 direct war-affected region, n=1303 less directly affected regions), 11-7 years, self-report | School survey |
| Thordardottir et al. (2020) | Cohort | 1991-2010, Balkan wars  Intrastate; one-sided violence | European non-war migrants and war migrants exposed to Balkan wars, N=252,200 (n=104,770 war migrants and n=147,430 non-war migrants), 0-19 years, Cause of Death Register | Swedish Cause of Death Register |
| Arënliu. (2014) | Cross-sectional | 2007, Kosovo war (1988-1989)  Intrastate; one-sided violence | Kosovar adolescents, N=2093, (M=17.14, SD=0.08), adolescents self-report | School survey |
| Morina, Lersner & Prigerson. (2011) | Cross-sectional | 2008-2009, Kosovo War 1998-1999  Intrastate | N=354 (n=179 bereaved, n=175 non-bereaved), (bereaved, M=20.3, SD=3.65, non-bereaved, M=20.0, SD=3.75), face-to-face interviews | Interview in community sample |
| Fajkic et al. (2010) | Time trend | 1986-1990 (prewar), 2002-2006 (postwar), Bosnian war 1992-1995  Intrastate; one-sided | Annualized data on suicide for children and adolescents, (N=135 prewar, N=65 postwar), 10-19 years, coroner's record | Prewar: Office for Statistics of the Republic of B&H, postwar: Ministry of Interior (Federation of B&H and Serb Republic) |
| Santic et al. (2010) | Time trend | 1984-2008, Bosnian war (1992-1995)  Intrastate; one-sided | Children who died by suicide in West-Herzegovina Canton, N=9 (10-19 years, total n=134), 10-19 years, coroners and parish priests | Statistic Institute of Federation of BiH, Public Health Institute of Federation of BiH and Cantonal Public Health Institute, Documents of Parish Priests |
| Mujkic et al. (2008) | Time trend | 1986-2005, Croatian Homeland war (1991-1995)  Intrastate; one-sided violence | Croatian children who died because of weapon-related injury, NA, 0-19 years, Croatian National Institute of Public Health | Vital Statistics Mortality Data from the Republic of Croatia Central Bureau of Statistics |
| Henderson et al. (2006) | Time trend | 1931-1952, World War II  Interstate | Data on suicide rates, NA, 15-24 years, N/A | The General Register Office for Scotland (GROS) records |
| Slodnjak, Kos & Yule. (2002) | Cross-sectional | 1994, Bosnian war (1992-1995)  Intrastate; one-sided | Bosnian refugee students and age-matched Slovenian students, N=265, 14-15 years, self-report | School survey |
| Murphy. (1986) | Cohort | 1921-1980, World War II (1939-1945)  Interstate | English and Welsh people who committed suicide, sample size NA, 15-19 years, coroners | Mortality Statistics of the Office of Population, Censuses and Surveys |
| **Middle East** | | | | |
| Alatrany et al. (2025) | Cross-sectional | Iraq conflicts, Interstate, intrastate; non-state, one-sided violence | School students, N=452, 13-17 years, self-report | Female schools from Karkh and Rusafa in Baghdad, two schools from each region |
| Al-Sammak & Al-Hamdany. (2025) | Cross-sectional | Iraq conflicts, Interstate, intrastate; non-state, one-sided violence | School, universities and public places, N=112, 15-19 years, self-report | Survey in School, universities, and public places |
| Rizk et al. (2023) | Cross-sectional | Syrian War  Interstate, intrastate, one-sided highly internationalized | Refugees, N=52, 14-21 years, interview | Interview in health centres |
| Dehnel et al. (2022) | Cross-sectional | 2018, Syrian civil war (ongoing)  Intrastate, one-sided; highly internationalized | Syrian refugee children and adolescents, N=339, 10-17 years, interview | Interview in community clinics and refugee centers |
| Hamdan & Hallaq (2021) | Cross-sectional | N/A, Israeli-Palestinian conflict (ongoing)  Extrasystemic; interstate; intrastate; non-state; one-sided violence | College and university students, N=303, 18-23 years, self-report | Surveys from public campuses at the West Bank in the Palestinian Authority |
| Chahine et al. (2020) | Cross-sectional | 2019, Lebanon war 2006, Lebanon conflict 2007 and 2008, Syrian civil war spillover in Lebanon 2011-2017  State-based; with interstate elements; one-sided violence | School students, N=1810, 14-17 years, self-report | School surveys from all Lebanese Mohafazat (Beirut, Mount Lebanon, North, South, and Bekaa) |
| Itani et al. (2017) | Cross-sectional | 2010, Israel-Palestinian conflict (ongoing)  Extrasystemic; interstate; intrastate; non-state; one-sided violence | Students living in the OPT as well as in United Nations Relief and Works Agency (UNRWA) camps in Gaza, the West Bank, Jordan, Lebanon, and Syria, N=14303, 13-15 years, self-report | Global school-based student health survey, schools, UNRWA in the West Bank |
| Rhiger, Elkit & Lasgaard. (2008) | Cross-sectional | 1998, Israel-Palestinian conflict (ongoing)  Extrasystemic; interstate; intrastate; non-state; one-sided violence | School students, N=494, 14-18 years (M=15.6 years, SD=0.7), self-report | Public and private school surveys |
| **North America** | | | | |
| Betancourt et al. (2017) | Cross-sectional | 2004-2010, War, terrorism or political violence outside of the US | Refugee-origin, immigrant-origin and U.S.-origin youth, N=343 (n=60 refugee-origin, n=143 immigrant-origin and n=140 U.S.-origin youth), mean age 13.07 years in refugee group, 13.26 years in immigrant group, 12.11 years in U.S.-origin group. CDS information was obtained from multiple respondents and sources by trained clinicians. | National Child Traumatic Stress Network’s Core Data Set (NCTSN CDS) |
| Betancourt et al. (2012) | Cross-sectional | N/A, refugee, asylum seeker, or immigrant child with a history of exposure to community violence | children, N=60, (mean=13.1, SD=4.13), self-report: interview | National Child Traumatic Stress Network’s Core Data Set (NCTSN CDS) |
| McCall. (1991) | Time trend | 1946-1986, World War II  Interstate | Data on suicide rates, NA,15-24 years, NA | Annual Vital Health Statistics Report, Volume II |
| **South and South-East Asia** | | | | |
| Jan et al. (2017) | Cross-sectional | N/A, Kashmir conflict (ongoing)Intrastate; entangled with interstate relations | Young adult and adolescent patients in a hospital, N=130, 15-24 years, interviews | Hospital-based study, Department of Psychiatry, Govt. Medical College, Srinagar, Jammu and Kashmir, India |
| Jegannathan & Kullgren. (2011) | Cross-sectional | N/A, Civil war against Khmer Rouge (ended in 1997)  Intrastate; one-sided | High school students, N=320, 15-18 years, self-report | Surveys in high schools in Cambodia |
| Kageyama & Naka. (1996) | Time trend | 1950-1990 (mainland Japan), 1960-1990 (Okinawa), World War II  Interstate | Data on suicide mortality, NA, 10-19 years, N/A | Annual statistics of Japan 1950-1990 and Okinawa 1960-1990 |
| Somasundaram & Rajadurai. (1995) | Time trend | 1980-1989, Sri Lanka, Civil War (1983-2009)  Intrastate; one-sided violence | Population of Jaffna town and surrounding suburbs during study period, NA, 15-24 years, coroner | Coroner's records from Jaffina Magistrate Courts |
| **South America** | | | | |
| Valencia et al. (2025) | Cross-sectional | Colombian conflict (ongoing)  Intrastate; non-state, one-sided violence | Internally displaced adolescents, N=135, Mean 14.4, Standard deviation (1.4), self-report | School survey |
| Ortega-Narvaez et al. (2024) | Cross-sectional | Colombia  Intrastate; non-state, one-sided violence | Adolescents in Cauca, N=977, <18 years, NA | Sistema Nacional de Vigilancia en  Salud Publica (SIVIGIL) Register |
| Moe at al. (2022) | Cross-sectional | 2018, Colombian conflict (ongoing)  Intrastate; non-state, one-sided violence | Adolescents from stratum representative of 170 municipalities, N=5209, 13-24 years, Self-report | Violence against children and youth survey (VACS) 2018 in Colombia |
| Marroquín et al. (2020) | Cross-sectional | 2015, Colombian conflict (ongoing)  Intrastate; non-state, one-sided violence | Subgroup of adolescents, N=1754, 12-17 years, Self-report | Colombian National Mental Health Survey (NMHS) 2015 |
| Orrego, Hincapié & Restrepo. (2020) | Cross-sectional | 2012, Colombian armed conflict (ongoing)  Intrastate; non-state, one-sided violence | Population residing in urban area, N=294,13-65 years, interview | Itagui mental health study |

M, mean; SD, standard deviation

# **Supplementary appendix Table 5: Summary of competed suicides, suicide attempts and suicidal ideation**

| **Author (Year)** | **Measures** | **Key findings** | **Risk factors** | **Protective factors** |
| --- | --- | --- | --- | --- |
| **Suicides** | | | | |
| **Europe** | | | | |
| Thordardottir et al. (2020) | International Statistical Classification of Diseases and Related Health Problems (ICD)-9, ICD-10 | Incidence rate (IR per 100,000 person-years) for suicide among 0-19 years was 2.52 (95% CI 1.50–4.26) for war migrants and 4.82 (2.90–7.99) for non-war migrants. | NA | NA |
| Fajkic et al. (2010) | Suicide rates before and after the war | Compared to the prewar period, the overall suicide rate decreased by 33.3% after the war. | NA | NA |
| Santic et al. (2010) | Suicide rates from records | Total number of suicides during the study period was 134, out of which 9 (6.7%) were committed by children between 10 and 19 years. 7 out of the 9 adolescent suicides were committed by males. | NA | NA |
| Mujkic et al. (2008) | Number of suicides from records | The weapon-related suicide rate was 0.51 per 100,000 prewar (1986-1990), 1.64 during the war (1991-1995), 1.59 early postwar (1996-2000) and 0.53 late postwar (2001-2005). Compared to prewar, the weapon-related suicide rate was 3.28 times higher during the war and 3.12 times higher early postwar. There was no significant difference between prewar and late postwar periods. There were no significant differences in non-weapon-related suicide rates. | NA | NA |
| Henderson et al. (2006) | The number of suicide deaths from annual records | Suicide rates among men aged 15-24 years rose during World War II, peaking during 1942 at 148 per million (41 deaths), then declined to 39 per million (10 deaths) by 1945. No significant change in suicide rates among women aged 15-24 years during the war. | NA | NA |
| Murphy. (1986) | Suicide rates from records | During World War II, the suicide rate for males (2.8) slightly fell compared to prewar period. In 1946-1955 the rate was high (3.7) but fell again in 1956-1960 (2.9). The rate then rose again in 1961-1965 (3.7). There wasn't much change in female suicide rates during the study period. | NA | NA |
| **South and South-East Asia** | | | | |
| Kageyama & Naka. (1996) | Annual suicide mortality | Suicide mortality in all sex-age-groups decreased dramatically in mainland Japan during the late 1950's and early 1960's. There was no evident decrease in suicide mortality in Okinawa during the early 1960's. Suicide mortality was significantly lower in Okinawa than in mainland Japan during the 1960's for males aged 10-19 and females regardless of age. | NA | NA |
| Somasundaram & Rajadurai. (1995) | Suicide rates from records | Before the war (1980-1982), the suicide rates were highest (62 per 100,000) for the age group 15-24. During the war (1983-1989) there was a very marked drop (240%) in suicide rates for the age group 15-24. | NA | NA |
| **North America** | | | | |
| McCall. (1991) | Annual U.S. suicide rates for the White male populations | Between 1946 and 1958 the 15-24 years white male suicide rate was quite stable, then climbed steeply between 1958 to 1977. Since 1977 the increasing trend continued at a slower pace until 1986. The 15-24 years male suicide rate increased from approximately 7 to 22 suicides per 100,000 between 1946 and 1986. | Dissolution of family, living in poverty | NA |
| **Suicide attempts** | | | | |
| **Africa** | | | | |
| Scharpf et al. (2024) | Mini-International Neuropsychiatric Interview for children (MINI-KID) | Past month prevalence of suicide attempts was 0.9% and the lifetime prevalence was 4.3%. | NA | NA |
| Kassa et al. (2023) | Composite international diagnostic interview (CIDI) | The prevalence of suicide attempts among high school students in Woldia town was 12.87%. | Depression and being female | Social support |
| Kinyanda et al. (2013) | “Have you ever attempted to take your life? (by ingesting poison, hanging, taking a drug overdose, drowning) in the previous 12 months and in your lifetime?” | Lifetime attempted suicide was 9.2% and 12-month attempted suicide was 2.6%. Significantly higher among females 101 (11.1%) than among males 43 (6.5%). | NA | NA |
| **Europe** | | | | |
| Sourander et al. (2024) | In the past six months, ‘Have you tried to commit suicide?’ | Girls living in the war-affected region reported significantly higher suicide attempts (9.5% vs 5.1%) compared to non war-region. | NA | NA |
| Thordardottir et al. (2020) | International Statistical Classification of Diseases and Related Health Problems (ICD)-9, ICD-10 | Incidence rate for suicide attempts among 0–19 years was 76.09 (69.14-83.73) for war migrants and 107.20 (96.27-119.37) for non-war migrants, crude HR for suicide attempts 0.57 (0.51-0.65) (p<0.001). | NA | NA |
| Arënliu. (2014) | Questionnaire on Suicide ideation and Behavior (Q-SIB) | Suicide attempts reported by 6.5% (4.1% of females and 2.4% of males). | Substance use, stressful events after war, suicide in family, self-blame, low levels of active coping and planning | Well-being, self-esteem |
|  |  | **Middle East** |  |  |
| Al-Sammak & Al-Hamdany. (2025) | Ask Suicide-  Screening Question | 13.8% (n=90) reported suicide attempts. | NA | NA |
| Ortega-Narvaez et al. (2024) | Registers | 32.3% reported suicide attempts. | relationship problems, a history of  psychiatric illness, and the use of psychoactive substance | NA |
| Itani et al. (2017) | "During the past 12 months, did you consider attempting suicide?” | The pooled prevalence of suicidal attempts was 19.9% in the past 12 months (19.3% in Gaza strip, 23.7% in West Bank, 18.5% in UNRWA Gaza, 21.7% in UNRWA Jordan, 14.6% in UNRWA Lebanon, 21.3% in UNRWA Syria and 21.5% in UNRWA West Bank). | NA | NA |
| Rhiger, Elkit & Lasgaard. (2008) | Trauma Symptoms Checklist (TSC) | 7.5% of the students reported suicide attempts within past two months. | NA | NA |
| **South America** | | | | |
| Moe et al. (2022) | ‘Have you ever tried to kill yourself?’ (asked only if responded yes to suicidal ideation) | Prevalence of suicide attempts was 10.1% in those exposed to community violence and 2.6% among those not exposed to community violence. | Exposure to direct or indirect home and/or community violence | NA |
| Marroquín et al. (2020) | Composite International Diagnostic Interview (CIDI-CAPI 3.0) | Suicide attempts over past 12 months significantly more common among displaced adolescents (9.2%) compared to non-displaced adolescents (2.1%) (p=0.008). | NA | NA |
| Orrego, Hincapié & Restrepo. (2020) | Composite International Diagnostic Interview (CIDI) | In the group with trauma related to the armed conflict, the prevalence of suicide attempts was 13.8% at age ≤13 years and 13.6% at age ≤14 years. | NA | NA |
| **South and South-East Asia** | | | | |
| Jan et al. (2017) | Clinical cases of suicide attempters in hospital | Among 15-24 years, 65% (n=130) attempted suicide. | NA | NA |
| Jegannathan & Kullgren. (2011) | Attitudes Toward Suicide (ATTS) | Of the total sample, 3.2% (n=10) (0.6% of the boys and 7.8% of the girls) attempted suicide attempts within the past year. | Exposed to suicides or attempts in family, partners or friends | NA |
| **Suicidal ideation** | | | | |
| **Africa** | | | | |
| Scharpf et al. (2024) | Mini-International Neuropsychiatric Interview for children (MINI-KID) | Past-month prevalence of suicidal ideation were 11.3%. The lifetime prevalence rates of suicidal ideation were 4.7% (n = 57). | Older age, post-traumatic stress disorder symptoms, internalizing, externalizing problem | NA |
| Kassa et al. (2023) | Composite international diagnostic interview (CIDI) | The prevalence of suicidal ideation among high school students in Woldia town was 16.3%. | Posttraumatic stress disorder, family history of suicide, anxiety, depression | Social support |
| Olema et al. (2014) | Mini International Neuropsychiatric Interview for Children and Adolescents (MINI-KID) | Children-report, mean of MINI suicidal ideation severity was 3.34 (SD=6.91) among abducted and 1.66 (SD=5.43) among non-abducted. Parents report-abducted 1.57 (5.0) and non-abducted 1.76 (4.21). | NA | NA |
| Okello et al. (2013) | Youth Self Report (YSR) (“I think about killing myself) | Of the total sample, 17.97% reported suicidal ideation, more commonly females (26.22%) than males (10.21%) over the past six months. | NA | NA |
| **Europe** | | | | |
| Sourander et al. (2024) | In the past six months, ‘Have you thought about committing suicide?’ | Girls living in the war-affected region reported significantly higher suicidal ideation (39.3% vs 19.6%) compared to those living in the non-war region. | NA | NA |
| Arënliu. (2014) | Questionnaire on Suicide Ideation and Behavior (Q-SIB) | Prevalence of suicidal ideation was 17.4% (11.5% in females and 5.9% in males). | Substance use, stressful events after war, suicide in family, self-blame, low levels of reported well-being, low levels of active coping and planning | Well-being, self-esteem |
| Slodnjak, Kos & Yule. (2002) | Modified War Trauma Questionnaire (WTQ) | The frequency of suicidal ideation was similar among the refugee students (24.14%) and the Slovenian students (26.15%). Direct intention to kill oneself was expressed by only 2 of the 265 refugees (0.77%) opposed to 5 of the 195 Slovenian students (2.56%). | NA | NA |
| **Middle East** | | | | |
| Alatrany et al. (2025) | Suicidal Ideation Attibues Scale (SIDAS), PHQ-p | Mean scores for suicide ideation on the SIDAS were 8.45 (SD = 10.30). 48.7% (n=220) reported they thought would be better off dead nearly every day in the last weeks. | Depression, quality of life, hopelessness | NA |
| Al-Sammak & Al-Hamdany. (2025) | Ask Suicide-  Screening Question | 25.5% (n=166) experienced suicidal ideation. | NA | NA |
| Rizk et al. (2023) | HEEADSSS: ‘Have you thought of hurting yourself or someone else’? | Out of adolescents screened positive for depression (n=32), 19 (59.38%) reported suicidal thoughts. | NA | NA |
| Dehnel et al. (2022) | Children’s Depression Inventory 2: (CDI-2) | 27.8% of children had suicidal ideation in the past two weeks. | NA | NA |
| Hamdan & Hallaq (2021) | Suicidal Behaviours Questionnaire-Revised (SBQ-R) | 20.4% (n=62) reported suicidal ideation within last 12 months within last 12 months**.** | Depressive symptoms, posttraumatic symptoms, sleep problems | NA |
| Chahine et al., (2020) | Columbia-suicide Severity Rating Scale (C-SSRS) | Prevalence of suicidal ideation since the last visit was 28.9% in adolescents. | Higher psychological abuse, child physical abuse, alcohol dependence, social fear, victimization/bullying score, impulsivity and internet addiction scores being female, separated parents, higher age | NA |
| Itani et al. (2017) | Suicidal ideation: ‘During the past 12 months did you ever seriously consider attempting suicide?’ | In the past 12 months, the pooled prevalence of suicidal ideation was 25.6% (25.9% in Gaza Strip, 28.5% in West Bank, 24.7% in UNRWA Gaza, 27.0% in UNRWA Jordan, 19.9% in UNRWA Lebanon, 26.8% in UNRWA Syria and 27.2% in UNRWA West Bank). | Marijuana use, having no close friends, tobacco use, loneliness, worry-induced insomnia, food insecurity, being the victim of a bully, being involved in physical fights and attacks, skipping school | Parental support |
| **South and South-East Asia** | | | | |
| Jegannathan & Kullgren. (2011) | Attitudes Toward Suicide (ATTS) | Of the total sample 11.0% (n=35) reported suicidal ideation within the past year. Of the boys 9.6% and 12.3% of the girls reported suicidal ideation within the past year, no significant difference between the genders. | Exposed to suicides or attempts in family, partners or friends | NA |
| **South America** | | | | |
| Valencia et al. (2025) | Positive and Negative Suicidal Ideation Scale for suicidal ideation (PANSI) | Mean of suicidal ideation 18.0 (SD=9.3). | NA | NA |
| Ortega-Narvaez et al. (2024) | Registers | 8.7% had suicidal ideation. | NA | NA |
| Moe et al. (2022) | ‘Have you ever thought about killing yourself?’ | Prevalence of suicide attempts was 15.4% in those exposed to community violence and 6.4% among those not exposed to community violence. | Exposure to direct or indirect home and/or community violence | NA |
| Marroquín et al. (2020) | Composite International Diagnostic Interview (CIDI-CAPI 3.0) | Suicidal ideation over past 12 months was significantly more common among displaced adolescents (19.8%) than non‑displaced adolescents (5.8%). | NA | NA |
| Orrego, Hincapié & Restrepo. (2020) | Composite International Diagnostic Interview (CIDI) | In the group with trauma related to the armed conflict, the prevalence of suicidal ideation was 34.5% at age ≤13 years and 29.5% at age ≤14 years. | NA | NA |
| **Suicide attempts or suicidal ideation** | | | | |
| **Africa** | | | | |
| Wolde & Dessalegn. (2022) | World Health Organization suicidal behavior assessment | Suicidal ideation or attempt reported by 15.11% (n=60). | PTSD | NA |
| Okello, Onnen & Musisi. (2007) | Mini International Neuropsychiatric Interview for Children and Adolescents (MINI-KID) | Past suicide attempts or suicidal ideation was more common among formerly abducted children (45%) than non-abducted children (23.2%) (p= 0.004). | NA | NA |
| **Europe** | | | | |
| Morina et al. (2011) | Mini International Neuropsychiatric Interview (MINI) | Current overall suicide attempts or suicidal ideation in bereaved group was 11.7% (n=21) and 6.3% (n=11) in the non-bereaved group. No significant difference between the two groups was found (p=0.074). | Prolonged grief disorder | NA |
| **North America** | | | | |
| Betancourt et al. (2017) | Clinical evaluation | Suicide attempts or suicidal ideation among refugee youth was 5.4%, 15.6% among immigrant youth and 9.3%. among U.S.-origin youth | NA | NA |
| Betancourt et al. (2012) | Clinical evaluation | 1.8% of war-affected children reported suicide attempts or suicidal ideation. | NA | NA |

NA, Not Assessed

# **Supplementary appendix Table 6. Quality assessment of the studies included in the review**

|  | **Q1** | **Q2** | **Q3** | **Q4** | **Q5** | **Q6** | **Q7** | **Q8** | **Q9** | **Q10** | **Q11** | **Q12** | **Q13** | **Total score** | **Percentage (%)** |
| --- | --- | --- | --- | --- | --- | --- | --- | --- | --- | --- | --- | --- | --- | --- | --- |
| Altrany et al. (2025) | 1 | 3 | 3 | 3 | 3 | 3 | 3 | 3 | 2 | 3 | 3 | 0 | 3 | 33 | 84.62 |
| Al-Sammak et al. (2025) | 0 | 3 | 2 | 2 | 2 | 2 | 2 | 3 | 2 | 1 | 2 | 0 | 0 | 21 | 53.85 |
| Ortega-NaNarváez et al. (2024) | 1 | 3 | 3 | 3 | 3 | 2 | 3 | 2 | 1 | 1 | 3 | 0 | 3 | 29 | 74.36 |
| Valencia et al., (2025) | 1 | 3 | 3 | 3 | 2 | 3 | 3 | 3 | 1 | 1 | 3 | 0 | 3 | 29 | 74.36 |
| Scharpf et al. (2024) | 3 | 3 | 3 | 3 | 2 | 3 | 3 | 3 | 2 | 1 | 3 | 2 | 2 | 33 | 84.62 |
| Sourander et al. (2024) | 2 | 3 | 3 | 3 | 2 | 3 | 3 | 3 | 3 | 2 | 3 | 0 | 3 | 33 | 84.62 |
| Kassa et al. (2023) | 3 | 3 | 3 | 3 | 3 | 1 | 3 | 2 | 2 | 1 | 3 | 0 | 2 | 29 | 74.36 |
| Rizk et al.  (2023) | 1 | 1 | 3 | 3 | 2 | 3 | 3 | 3 | 2 | 2 | 3 | 2 | 3 | 31 | 79.49 |
| Dehnel, et al., (2022) | 1 | 1 | 3 | 3 | 0 | 1 | 3 | 3 | 1 | 3 | 3 | 0 | 2 | 24 | 61.54 |
| Moe et al., (2022) | 3 | 3 | 3 | 3 | 3 | 3 | 3 | 3 | 3 | 2 | 3 | 3 | 3 | 38 | 97.44 |
| Wolde & Dessalegn (2022) | 1 | 1 | 3 | 3 | 3 | 2 | 3 | 3 | 3 | 2 | 3 | 0 | 2 | 29 | 74.36 |
| Hamdan & Eyad, (2021) | 3 | 3 | 3 | 3 | 2 | 3 | 3 | 3 | 1 | 3 | 3 | 0 | 2 | 32 | 82.05 |
| Chahine, et al., (2020) | 3 | 3 | 3 | 3 | 3 | 3 | 3 | 2 | 3 | 2 | 3 | 0 | 2 | 33 | 84.62 |
| Marroquín Rivera et al., (2020) | 1 | 1 | 3 | 3 | 2 | 3 | 3 | 2 | 3 | 2 | 3 | 0 | 3 | 29 | 74.36 |
| Orrego, Hincapie & Restrepo (2020) | 1 | 1 | 3 | 3 | 3 | 3 | 3 | 1 | 1 | 2 | 3 | 0 | 2 | 26 | 66.67 |
| Thordardottir et al., (2020) | 1 | 1 | 3 | 3 | 3 | 3 | 3 | 3 | 3 | 3 | 3 | 0 | 3 | 32 | 82.05 |
| Betancourt, et al., (2017) | 1 | 1 | 3 | 3 | 3 | 3 | 3 | 3 | 3 | 3 | 3 | 0 | 3 | 32 | 82.05 |
| Itani, Jacobsen & Kraemer (2017) | 3 | 3 | 3 | 3 | 2 | 2 | 3 | 3 | 3 | 3 | 3 | 3 | 3 | 37 | 94.87 |
| Jan et al., (2017) | 3 | 3 | 3 | 3 | 0 | 2 | 3 | 1 | 1 | 1 | 2 | 0 | 2 | 24 | 61.54 |
| Arenliu (2014) | 3 | 3 | 3 | 3 | 3 | 2 | 3 | 3 | 3 | 0 | 3 | 0 | 2 | 31 | 79.49 |
| Olema et al., (2014) | 1 | 1 | 3 | 3 | 2 | 3 | 3 | 3 | 1 | 2 | 3 | 0 | 3 | 28 | 71.79 |
| Kinyanda et al., (2013) | 3 | 3 | 3 | 3 | 3 | 3 | 3 | 3 | 3 | 2 | 3 | 0 | 3 | 35 | 89.74 |
| Okello et al., (2013) | 3 | 1 | 3 | 3 | 2 | 2 | 3 | 3 | 2 | 3 | 3 | 0 | 2 | 30 | 76.92 |
| Betancourt et al., (2012) | 0 | 0 | 3 | 0 | 3 | 0 | 0 | 3 | 2 | 0 | 3 | 2 | 3 | 19 | 48.72 |
| Jegannathan & Kullgren (2011) | 3 | 3 | 3 | 3 | 2 | 3 | 3 | 2 | 3 | 3 | 3 | 0 | 2 | 33 | 84.62 |
| Morina, von Lersner & Prigerson (2011) | 2 | 1 | 3 | 3 | 2 | 3 | 3 | 3 | 3 | 2 | 3 | 0 | 3 | 31 | 79.49 |
| Fajkic et al., (2010) | 2 | 3 | 3 | 3 | 3 | 3 | 3 | 3 | 3 | 0 | 3 | 0 | 2 | 31 | 79.49 |
| Santic et al. (2010) | 2 | 3 | 1 | 3 | 0 | 1 | 3 | 2 | 1 | 1 | 3 | 0 | 0 | 20 | 51.28 |
| Mujkic et al., (2008) | 1 | 1 | 3 | 3 | 3 | 3 | 3 | 3 | 3 | 1 | 3 | 0 | 2 | 29 | 74.36 |
| Rhiger, Elkit & Lasgaard (2008) | 1 | 1 | 3 | 3 | 2 | 3 | 3 | 2 | 1 | 0 | 2 | 0 | 3 | 24 | 61.54 |
| Okello, Onen & Musisi (2007) | 1 | 1 | 3 | 3 | 1 | 3 | 3 | 3 | **2** | 3 | 3 | 0 | 3 | 29 | 74.36 |
| Henderson, et al., (2006) | 3 | 3 | 3 | 3 | 3 | 3 | 3 | 3 | 3 | 1 | 3 | 0 | 1 | 32 | 82.05 |
| Slodnjak, Kos & Yule (2002) | 1 | 1 | 3 | 3 | 2 | 3 | 3 | 3 | 3 | 0 | 2 | 0 | 2 | 26 | 66.67 |
| Kageyama & Naka (1996) | 3 | 3 | 3 | 3 | 3 | 3 | 3 | 3 | 3 | 3 | 3 | 0 | 0 | 33 | 84.62 |
| Somasundaram & Rajadurai (1995) | 3 | 3 | 3 | 3 | 3 | 3 | 3 | 3 | 3 | 1 | 3 | 0 | 3 | 34 | 87.18 |
| McCall, (1991) | 3 | 3 | 3 | 3 | 3 | 3 | 3 | 3 | 3 | 3 | 3 | 0 | 0 | 33 | 84.62 |
| Murphy, (1986) | 3 | 3 | 3 | 3 | 3 | 2 | 3 | 2 | 3 | 1 | 3 | 0 | 0 | 29 | 74.36 |
| **Total scores** | 68 | 68 | 97 | 96 | 76 | 84 | 96 | 88 | 79 | 58 | 96 | 12 | 71 | 989 |  |

Summary of risk of bias assessment of 32 included studies using Quality Assessment Tool for Studies with Diverse Designs (QuADs): Q1Theoretical or conceptual underpinning to the research, Q2 Statement of research aim/s, Q3 Clear description of research setting and target population, Q4 The study design is appropriate to address the stated research aim/s, Q5 Appropriate sampling to address the research aim/s, Q6 Rationale for choice of data collection tool/s, Q7 The format and content of data collection tool is appropriate to address the stated research aim/s, Q8 Description of data collection procedure, Q9 Recruitment data provided, Q10 Justification for analytic method selected, Q11 The method of analysis was appropriate to answer the research aim/s, Q12 Evidence that the research stakeholders have been considered in research design or conduct, Q13 Strengths and limitations critically discussed.
