# Supplementary material for: Suicides, suicide attempts and suicidal ideation among children and young people exposed to war: a scoping review
Source: BMJ Open. 2026 Jun 4;16(6):e103126. doi: 10.1136/bmjopen-2025-103126 (PMC13239371; doi:10.1136/bmjopen-2025-103126)
Supplement: Supplementary file 2 [file bmjopen-2025-103126.reviewer_comments.pdf]

PEER REVIEW HISTORY

BMJ Open publishes all reviews undertaken for accepted manuscripts. Reviewers are asked to complete a checklist review form and are provided with free text boxes to elaborate on their assessment. These free text comments are reproduced below.

ARTICLE DETAILS

Title (Provisional)

Suicides, suicide attempts and suicidal ideations among children and young people exposed to war: A scoping review

Authors

Silwal, Sanju; Westerlund, Minja; Wan Mohd Yunus, Wan Mohd Azam; Jaakola-Siimes, Saara; Klomek, Anat Brunstein; Sourander, A.

VERSION 1 - REVIEW

**Reviewer**	**1**
**Name**	**Wilson-Lemoine, Emma**
**Affiliation**	**University of Virginia, Psychology**
**Date**	**30-Jun-2025**
**COI**	**None**

Thank you for submitting this protocol. It is a very important, and timely, topic and I think this has good potential. Most of my comments refer to aspects of the PRISMA checklist that need to be clarified and reported, and structure in the Results and Discussion. A thorough proof reading would also be helpful.

Abstract

Design

1. Make clear that "suicides" refers to suicide deaths or completed suicides. Do this throughout the paper.

Methods:

1. Check the Abstract contains all aspects of the revised 2020 guidelines for PRISMA abstracts - https://www.bmj.com/content/372/bmj.n160 (see Item 2, Box 2)
    a. E.g., "Specify the methods used to present and synthesise results (item #6)"
    b. Item #3 – specify that only quantitative studies are included
    c. Item #5 – specify the methods used to assess risk of bias in the included studies
    d. Item #12 – specify OSF number/add link

Results:

1. What is the extent of suicidal thoughts and behaviors among children and young people? Some global statistics would be helpful.
2. It would be helpful to state how many studies were reported for each outcome (i.e., suicide deaths (n = X), suicide attempts (n = X), suicidal ideation (n = X))
3. Saying “most of the research was conducted in European countries” is slightly misleading because it was 10 out of 22 included studies. Consider a way to rephrase such as “Included studies spanned six continents, from Latin America (n=3, Colombia only) to Europe (n=10).”
4. I think it would be helpful to mention that you looked at suicidal outcomes during and post war, and also a sentence about timeframes: e.g., how many of the included studies were wars/conflicts in the past 2 decades (vs. WWII).

Conclusion:

1. Change to ‘**suicidal thoughts** and behaviors’

Introduction

- It would be helpful to add a brief sentence defining the difference between war and armed conflict. It would then be useful to put that into context – for example, how many wars/armed conflicts are estimated to have occurred since WWII and/or in recent decades?

Methods:

PRISMA-specific considerations (given that you state “This review reporting follows the PRISMA guidelines(16)”):

1. Check the paper adheres to changes to the 2020 version as your current citation is for 2009. Details of the updates are found here: https://www.bmj.com/content/372/bmj.n71 and https://www.bmj.com/content/372/bmj.n160
2. More details should follow “This review reporting follows the PRISMA guidelines(16)”. Signpost to the full search strategy (either signpost to the LSR or attach as supplementary materials). Alternatively, attach the PRISMA checklist and signpost reader to where they can find information about each of the 27 criteria.
3. See PRISMA 2020 guidance which suggests shifting away from reporting on ‘quality assessment’ to ‘risk of bias’ and note difference with ‘assessing certainty’ (see items 11; 15 of 2020 checklist).
4. How did you meet Item #15 (‘certainty assessment’)? You state that you generated “a consensus rating (i.e., whether the study was of reasonable quality or not)” – did you use a tool for this? What were the criteria when assessing quality? See BMJ papers for more details of this. They provide GRADE as an example tool.

Exclusion criteria:

1. I assume mixed methods studies were included as long as quantitative findings were reported. If so, I’d rephrase to ‘Studies that **only** reported qualitative findings’

Search strategy and selection criteria

1. “Searches were conducted for studies published up to 17 March 2024. Searches were not limited by publication date or geographic location”. – I’m not sure what is meant. By ‘not limited by publication date’ – given that you stated the searches were conducted from

inception until 17 March 2024, I think you can remove this and just say "Searches were not limited by geographic location". The same comment applies to the Abstract.

2. Specify how decisions were made if there were a study with a mixed sample of ages (e.g., up to 25, or up to 30 years).
3. See criteria for Item 8 "Essential elements for systematic reviews regardless of the selection processes used" (p7) of PRISMA article Page et al., (2021): https://www.bmj.com/content/372/bmj.n160
    a. How many reviewers screened each record? Were all papers double screened?
4. See criteria for Item 9 in "Essential elements" (p9-10) from Page et al., (2021)
    a. Was data extraction undertaken independently by one or more researchers? Were all records double screened? Or only a certain % cross-checked? Specify, and if not 100% double checked, explain how error in data extraction was minimised.
    b. "Report any processes used to obtain or confirm relevant data from study investigators (such as how they were contact, what data were sought, and success in obtaining the necessary information"
5. Explain what is meant by "A checkbox table was used to depict comparisons between the included studies."
6. See criteria for Item 10a in "Essential elements" (p10-11) of Page et al., (2021)
    a. "List and define the outcome domains and time frame of measurement for which data were sought"
        i. Given that some studies (e.g., those in the UK) measure self-harm, which can include self-injury with suicidal intent, were these studies within the scope of your review? From the search strategy it seems yes. Outlining the scope of your outcomes would be helpful. See example on page 11 of the Page et al., 2021 article to help.
        ii. Did studies need to demonstrate that the suicidal thoughts and behaviors occurred during or after the period of war or armed conflict?
7. See criteria for Item 11 "Risk of bias assessment"
    a. See Box 4 – Item 11 in PRISMA focuses on risk of bias rather than "quality" (which may encompass constructs beyond those that bias the findings). Rephrase this section to "risk of bias" assessment and make clear how your assessment focuses on *bias* – specifying the domains/components of the QuADS will help with this. If QuADS includes domains that focus on quality > bias (i.e., "imprecision, reporting completeness, ethics, and applicability"), explain and demonstrate that papers given more weight in the paper are those with low *bias* (not *quality* more broadly).
    b. "Specify the methodological domains/components/items of the risk of bias tool used"
8. Item 13c PRISMA – describe methods to tabulate or visually display results. Explain how results will be presented and synthesised.

Other:

1. Make clear how Items 11 (risk of bias) and 15 (certainty assessment) are addressed in your paper. Often, two different tools will be used for the two different tasks. See Page et al., (2021) - https://www.bmj.com/content/372/bmj.n160 - for more information.
2. Search re-run: It will be appropriate to re-run the search from May 2024 (based on COCHRANE best practice): "*Rerun or update searches for all relevant sources within 12 months before publication of the review or review update, and screen the results for potentially eligible studies.*"
3. In the context of suicide, the phrase 'committed' has been used in the tables; it's not appropriate to say 'committed suicide' so please change this terminology.

Results

Characteristics

1. Some of the information in the first paragraph can be removed if the information is presented in the PRISMA flow diagram.
2. I think the references to Figures 2 and 3 have been mixed up.
3. Figure 3 is useful and could be described – i.e., which war/conflicts are represented in the papers the most? I think it would be helpful to differentiate studies by era/decade – how many papers reported results for war/conflict in the past 10-20 years?
4. Add a description with the range of participant numbers across the studies (e.g., Across all studies, participant numbers ranged from XX to YY).
5. PRISMA flow chart: Add appropriate citation for the flowchart (Page MJ, et al. BMJ 2021;372:n71. doi: 10.1136/bmj.n71)

Quality assessment

1. See comment above about risk of bias vs. quality assessment terminology and scope, as per PRISMA, and change wording accordingly.
2. Proof read and check phrasing: "Most studies did not use stakeholders in the research design or conduct pilot study influencing design or conduct of the study." – change "use" to "involve", clarify what is meant by conduct**ing** and pilot study…

Outcomes

1) At the end of your Introduction you say "we will identify the prevalence, risk and protective factors associated with suicides, attempts and suicidal ideations"
   a) Make sure the Results are structured in a way that match the aims as set out in the Intro and Methods
2) I think it would be clearer to have separate headings for 'suicide deaths', 'suicide attempts', 'suicidal ideation'. Then, within each of these, explore 1) prevalence and 2) associated risk and protective factors
3) Also, you mention 'suicide plan' for the first time in the manuscript – it would be helpful to explain in the methods how this fits with the 3 outcomes that you aim to explore.
4) *Suicide attempts and suicidal ideation and mental health* is a confusing heading. See my suggestion in *Outcomes* 1a.

Tables

- Some further structure could be added into the tables (it's unclear why they aren't in alphabetical order, too)
  - For table 2, under the three outcome sections/headings, could there be further sub-headings with results organised by continent?

Discussion

- In the first sentence, make sure you are clear about the purpose of this review. Specifically, "the prevalence, risk and protective factors associated with suicides, attempts and suicidal

ideations" – it's not clear in this first paragraph that you are looking at risk and protective factors.

- Differentiating between suicide rates during and post war is helpful
- In general, try to structure based on your main aims (1. Prevalence; 2. Risk and protective factors) and will some structure when describing your results with reference to the literature based on the 3 outcomes. At times, it feels like there isn't a clear structure.
- The section on limitations is too brief and warrants a paragraph of its own
- The discussion about future research is a sentence of the end of the Conclusion, which isn't enough and also warrants it's own section or to be integrated throughout the Discussion.

**Reviewer**	**2**
**Name**	**Auby, Philippe**
**Affiliation**	**CoBTeK lab**
**Date**	**19-Aug-2025**
**COI**	**None**

Thank you very much for giving me the opportunity to review this interesting manuscript, and please find my comments below:

Abstract:

Would "More than half a billion…" be a more impactful wording than "Millions of children and young people live in areas affected by war"?

According to UNICEF's Humanitarian Action for Children 2025, at least 460 million children are affected. In addition, as this report does not include youngsters between 18 and 24 years, the wording could be adjusted to provide a better sense of the magnitude of the issue.

https://www.unicef.org/reports/humanitarian-action-children-2025-overview

I believe you should further develop the 'Study Limitations' section.

-For instance, should at least a third, more specific limitation be added with respect to the underrepresentation of some major conflict zones, namely Africa or the Middle East, where larger populations of children and young adults are affected by ongoing wars?

-Should the first limitation you suggested and this potential third one also be considered as an opportunity to call for a future, larger "multilingual" review, and ultimately be mentioned in the discussion or conclusion?

I wonder if the "other" reasons for excluding 7 studies should not be specified and reported, at the very least, on page 10 and possibly as a footnote to Figure 1.

The reviewed studies span a significant period. You mentioned on page 5 that historical factors can play a role in human reactions to catastrophes: have you found any hints that suicide risk among war-affected children and young people could vary across historical eras?

I agree that the "results regarding refugees should hence be interpreted with caution ». Furthermore, as you pointed out, refugee studies are heterogeneous, conducted either in refugee camps close to the war zones or in third countries where people are seeking asylum. You might want to consider expanding the paragraph on page 16 about refugees and eventually explaining your rationale for including those studies. These studies might not be a perfect "proxy", however, they are informative.

Minor points:

-PTSD is never spelt out as Post Traumatic Stress Disorder. I would suggest doing this on either page 4 or 13.

-Define what a "convenient sampling" is.

VERSION 1 - AUTHOR RESPONSE

Reviewer: 1
Dr. Emma Wilson-Lemoine, University of Virginia, King's College London
Comments to the Author:
Thank you for your paper and work on this important topic.
Thank you for submitting this protocol. It is a very important, and timely, topic and I think this has good potential. Most of my comments refer to aspects of the PRISMA checklist that need to be clarified and reported, and structure in the Results and Discussion. A thorough proof reading would also be helpful.
Abstract
Design
1. Make clear that "suicides" refers to suicide deaths or completed suicides. Do this throughout the paper.
Response: Thank you for your comments. We have added text in the manuscript and revised the term throughout the manuscript.
''Suicides referred to suicide deaths or completed suicides caused by self-directed injurious behavior with intent to die as a result of the behavior.[26] '' (Page 9, Methods)

Methods:
1. Check the Abstract contains all aspects of the revised 2020 guidelines for PRISMA abstracts -
https://www.bmj.com/content/372/bmj.n160 (see Item 2, Box 2)
a. E.g., "Specify the methods used to present and synthesise results (item #6)"
b. Item #3 – specify that only quantitative studies are included
c. Item #5 – specify the methods used to assess risk of bias in the included studies
d. Item #12 – specify OSF number/add link

Response: We modified the manuscript as a 'scoping review' and revised it based on (PRISMA-ScR) Checklist. We have added information in the abstract and also in the method section.
''The quality of the included articles was assessed using Quality Assessment with Diverse Studies (QuADS). The protocol of the review was registered at the Open Science Framework on 29 March 2022 (https://osf.io/7kszh/).'' (Abstract)
''The scoping review was conducted in accordance with the Preferred Reporting Items for Systematic Reviews and Meta-Analyses extension for scoping reviews (PRISMA-ScR).[24]'' **(Page 7, Methods)**

Results:
1. What is the extent of suicidal thoughts and behaviors among children and young people? Some global statistics would be helpful.
Response: We have added text in the abstract.
''Suicide remains one of the third leading cause of death worldwide among aged 15-29 years.'' (Abstract)
''Suicide and suicidal behaviors are global public health concerns. Suicide remains one of the third leading cause of death worldwide among aged 15-29 years.[11] Suicide attempts, and ideation are antecedents of suicides in children and young people.[12]'' (Introduction, Page 5)

2. It would be helpful to state how many studies were reported for each outcome (i.e., suicide deaths (*n* =X), suicide attempts (*n* = X), suicidal ideation (*n* = X))
Response: We have included information on outcomes in the abstract.
''The reported outcomes were suicides (n=9), suicide attempts (n=15), suicidal ideation (n=21).'' **(Abstract)**

3. Saying "most of the research was conducted in European countries" is slightly misleading because it was 10 out of 22 included studies. Consider a way to rephrase such as "Included studies spanned six continents, from Latin America (n=3, Colombia only) to Europe (n=10)."
Response: We revised the manuscript.
''Included studies spanned six continents, from Latin America (n=5, Colombia only) to Europe (n=10).'' (Abstract)

4. I think it would be helpful to mention that you looked at suicidal outcomes during and post war, and also a sentence about timeframes: e.g., how many of the included studies were wars/conflicts in the past 2 decades (vs. WWII).
Response: We agree that including the timeframes would increase clarity for the readers. We have added the following sentence in the abstract.
''Most studies (> 20) focused on conflicts ongoing during or until the 2000s, whereas only 3 focused on World War II.'' (Abstract)

Conclusion:
1. Change to '**suicidal thoughts** and behaviors'
Response: We have added text in the abstract.

***‘‘There is a need for more methodologically consistent and rigorous research on suicidal thoughts and behaviors in children and young people exposed to war or armed conflicts.’’ (*Abstract)**

Introduction

• It would be helpful to add a brief sentence defining the difference between war and armed conflict. It would then be useful to put that into context – for example, how many wars/armed conflicts are estimated to have occurred since WWII and/or in recent decades?

Response: We have added text in the revised manuscript.

***‘‘In 2024, the number of armed conflicts rose worldwide with 61 active conflicts involving at least one state. Of these, 11 escalated to the level of war, defined as a conflict resulting at least 1000 battle-related deaths in a year.’’* (Introduction, Page 6)**

Methods:

PRISMA-specific considerations (given that you state “This review reporting follows the PRISMA
guidelines(16)”):

1. Check the paper adheres to changes to the 2020 version as your current citation is for 2009. Details of the
updates are found here: https://www.bmj.com/content/372/bmj.n71 and https://www.bmj.com/content/372/bmj.n160

Response: We revised the manuscript as a scoping review. The revised manuscript adheres to the PRISMA-ScR guidelines.

2. More details should follow “This review reporting follows the PRISMA guidelines(16)”. Signpost to the full search strategy (either signpost to the LSR or attach as supplementary materials). Alternatively, attach the PRISMA checklist and signpost reader to where they can find information about each of the 27 criteria.

Response: Thank you. We have included search strategy and PRISMA checklist in the supplementary.

3. See PRISMA 2020 guidance which suggests shifting away from reporting on ‘quality assessment’ to ‘risk of bias’ and note difference with ‘assessing certainty’ (see items 11; 15 of 2020 checklist).

Response: Thank you for the comment. We have revised the review as a scoping review based on the Editor’s comment. Given that scoping reviews are generally conducted to provide an overview of existing evidence, we have not performed a risk of bias assessment, but assessed the quality of each article using the Quality Assessment with Diverse Studies (QuADS).

4. How did you meet Item #15 (‘certainty assessment’)? You state that you generated “a consensus rating (i.e., whether the study was of reasonable quality or not)” – did you use a tool for this? What were the criteria when assessing quality? See BMJ papers for more details of this. They provide GRADE as an example tool.

Response: We used the Quality Assessment with Diverse Studies (QuADS) to score each included article. The mean scores for each item were then calculated to identify

deficiencies in the study quality. Findings from higher quality papers were given more weight in the discussion of the paper.

Exclusion criteria:
1. I assume mixed methods studies were included as long as quantitative findings were reported. If so, I'd rephrase to 'Studies that **only** reported qualitative findings'
Response: We have modified text in the revised manuscript:
''Studies were excluded if studies were: 1) conference abstracts, case reports, reviews, only qualitative study or case series, 2) age of the participants more than 24 years, and 3) language of the study is not English.'' (Page 8, Methods)

Search strategy and selection criteria
1. "Searches were conducted for studies published up to 17 March 2024. Searches were not limited by publication date or geographic location". – I'm not sure what is meant. By 'not limited by publication date' – given that you stated the searches were conducted from inception until 17 March 2024, I think you can remove this and just say "Searches were not limited by geographic location". The same comment applies to the Abstract.
Response: We updated our search until November 2025 modified the text in the revised manuscript.
''A scoping review of studies was conducted using Web of Science, PubMed, Embase and PsychINFO databases from their inception to November 2025, without any restrictions on geographic location.'' (Abstract)

''Searches were not limited by geographic location.'' (Page 7, Methods)

2. Specify how decisions were made if there were a study with a mixed sample of ages (e.g., up to 25, or up to 30 years).
Response: Thank you for your important comment. If a study included participants age group >24 years, but reported separate results for the age group <24 years, we included it in our review. We had a flexibility of ± 1 year age. We have added that text in the revised manuscript.
''The inclusion criteria for the review were: 1) observational studies including retrospective, prospective, cross-sectional, longitudinal, case-control, and cohort studies, 2) studies reported any suicides, suicide attempts and suicidal ideations of children and young people aged 0-24 years exposed to war, with a flexibility of ± 1 years (studies including participants above this age group were included if results were reported separately).'' (Methods, Page 8)

3. See criteria for Item 8 "Essential elements for systematic reviews regardless of the selection processes used" (p7) of PRISMA article Page et al., (2021): https://www.bmj.com/content/372/bmj.n160
a. How many reviewers screened each record? Were all papers double screened?
Response: Title and abstract, as well as full-text screening of each article, were conducted by two reviewers.
***''Each article was screened by at least two reviewers.''* (Methods, Page 8)**

4. See criteria for Item 9 in “Essential elements” (p9-10) from Page et al., (2021)
a. Was data extraction undertaken independently by one or more researchers? Were all records
double screened? Or only a certain % cross-checked? Specify, and if not 100% double checked,
explain how error in data extraction was minimised.
Response: The data extraction was independently done by two reviewers. All records were double screened and if there was any disagreement, final decisions were made after discussing with a senior researcher.
‘‘For studies identified in our search, at least two reviewers independently extracted the relevant data. Discrepancies were resolved in consultation with a third reviewer (MS).’’ **(Methods, Page 8-9)**

b. “Report any processes used to obtain or confirm relevant data from study investigators (such as
how they were contact, what data were sought, and success in obtaining the necessary information”.
Response: We contacted the corresponding authors if some information was missing in the articles. Reminder emails were sent a week after the first email. The information is provided in the supplementary materials.
‘‘We contacted the corresponding authors to obtain missing information, sending reminder emails one week later (see supplemental table 2). ’’ **(Methods, Page 8)**

5. Explain what is meant by “A checkbox table was used to depict comparisons between the included studies.”
Response: We created a table listing all studies assessed during the full-text screening, with checkbox-style indicators showing whether each study met the inclusion criteria. This format allowed us to visually compare studies across the same set of criteria and clearly identify which articles were included or excluded and for what reasons.

6. See criteria for Item 10a in “Essential elements” (p10-11) of Page et al., (2021)
a. “List and define the outcome domains and time frame of measurement for which data were sought”
Response: We added the definition and time measurement of the outcomes in the revised manuscript.
‘‘Suicide referred to suicide deaths or completed suicides caused by self-directed injurious behavior with intent to die as a result of the behavior. Suicide attempts were defined as self-directed, potentially injurious behaviors with intent to die. Suicidal ideation referred to thoughts of engaging in suicidal behavior. Data were included when these outcomes were measured during or after exposure to war or armed conflict.’’ (Methods, Page 9)

i. Given that some studies (e.g., those in the UK) measure self-harm, which can include self-injury with suicidal intent, were these studies within the scope of your review? From the search strategy it seems yes. Outlining the scope of your outcomes would be helpful. See example on page 11 of the Page et al., 2021 article to help.

Response: Thank you for this important question. We did not include studies that reported self-harm as an outcome when it was defined broadly to include both self-harm with or without suicidal intent. Because this broad definition does not allow us to isolate suicidal intent, such studies were excluded unless they clearly specified that the self-harm involved an intent to die.

ii. Did studies need to demonstrate that the suicidal thoughts and behaviors occurred during or after the period of war or armed conflict?
Response: To be eligible, studies needed to include participants who were exposed to war or armed conflicts, and the suicidal thoughts or behaviors assessed in those studies had to occur during or after that period of exposure. This ensured that the outcomes were related to the conflict context.

7. See criteria for Item 11 "Risk of bias assessment"
a. See Box 4 – Item 11 in PRISMA focuses on risk of bias rather than "quality" (which may encompass constructs beyond those that bias the findings). Rephrase this section to "risk of bias"
assessment and make clear how your assessment focuses on *bias* – specifying the domains/components of the QuADS will help with this. If QuADS includes domains that focus
on quality > bias (i.e., "imprecision, reporting completeness, ethics, and applicability"), explain
and demonstrate that papers given more weight in the paper are those with low *bias* (not *quality*
more broadly).
Response: Please see above response 3.
b. "Specify the methodological domains/components/items of the risk of bias tool used"
Response: Please see above response 3.

8. Item 13c PRISMA – describe methods to tabulate or visually display results. Explain how results will be presented and synthesised.
Response: We have added text in the revised manuscript.
''We organized results of the scoping review by various geographical regions, type of conflict and key characteristics of the included studies. The results were presented based on estimates in the included studies, e.g., prevalence of suicides, attempts and suicidal ideations and relevant risk and protective factors associated with the outcomes.'' (Methods, Page 9)

Other:
1. Make clear how Items 11 (risk of bias) and 15 (certainty assessment) are addressed in your paper. Often, two different tools will be used for the two different tasks. See Page et al., (2021) -
https://www.bmj.com/content/372/bmj.n160 - for more information.
Response: We revised the manuscript as a scoping review thus, risk of bias was not assessed.

2. Search re-run: It will be appropriate to re-run the search from May 2024 (based on COCHRANE best practice): "*Rerun or update searches for all relevant sources within 12 months before publication of the review or review update, and screen the results for potentially eligible studies.*"
Response: Thank you for your comment. We have updated the search and revised the manuscript based on the findings.

3. In the context of suicide, the phrase 'committed' has been used in the tables; it's not appropriate to say 'committed suicide' so please change this terminology.
Response: We have revised the term thoroughly in the manuscript.

Results
Characteristics
1. Some of the information in the first paragraph can be removed if the information is presented in the PRISMA flow diagram.
Response: We have made changes in the revised manuscript.

2. I think the references to Figures 2 and 3 have been mixed up.
Response: We have made changes in the revised manuscript.

3. Figure 3 is useful and could be described – i.e., which war/conflicts are represented in the papers the most? I think it would be helpful to differentiate studies by era/decade – how many papers reported results for war/conflict in the past 10-20 years?
Response: The included studies primarily assessed the Colombian conflict (n = 5) and the civilian war between Uganda and Kony's LRA Rebel group (n = 3). In the past 10–20 years, studies reported suicidal outcomes from the Palestinian-Israeli conflict, the Syrian civil war, Lebanon conflict, Colombian conflict, and the Russian invasion of Ukraine.
'' Most common types of conflict addressed in the studies were intrastate conflicts, of which most also included instances of one-sided violence (i.e. use of armed force against civilians by a government actor or formally organized group).[30], for example, Colombian conflict (n = 5) and the civilian war between Uganda and Kony's LRA Rebel group (n = 3). Interstate conflicts were addressed in studies relating to World War II and the recent Russia-Ukraine war, although some conflicts included interstate elements (e.g. conflicts in Lebanon). Geographically, these interstate studies were limited to Europe and the USA, whereas studies from intrastate conflict contexts were spread across all regions covered in the current review. The nature of these conflicts, and their spreading over time is in line with the scholarly consensus on that modern wars are more conducted within states than between states.[9] In the past 10–20 years, studies reported suicidal outcomes from the Palestinian-Israeli conflict, Syrian civil war, Lebanon conflict, Colombian conflict, and the Russian invasion of Ukraine.'' (Results, Page 11)

4. Add a description with the range of participant numbers across the studies (e.g., Across all studies, participant numbers ranged from XX to YY).
Response: We added text in the revised manuscript.

''The number of participants ranged from 52-252200, with most studies having sample sizes of less than 1,000.'' **(Results, Page 11)**

5. PRISMA flow chart: Add appropriate citation for the flowchart (Page MJ, et al. BMJ 2021;372:n71. doi: 10.1136/bmj.n71)
Response: We have added the citation of PRISMA flowchart of scoping review.

Quality assessment
1. See comment above about risk of bias vs. quality assessment terminology and scope, as per PRISMA, and change wording accordingly.
Response: We revised the manuscript as scoping review thus risk of bias was not assessed.

2. Proof read and check phrasing: "Most studies did not use stakeholders in the research design or conduct pilot study influencing design or conduct of the study." – change "use" to "involve", clarify what is meant by conducting and pilot study…
Response: We have added the text in the revised manuscript. As per QuaDs guideline item 12 'Evidence that the research stakeholders have been considered in research design or conduct', it is recommended stated that research are designed after consultation with stakeholders, for example conducting pilot study with feedback that influences the study design or by establishing project advisory group to guide the research.

Outcomes
1) At the end of your Introduction you say "we will identify the prevalence, risk and protective factors associated with suicides, attempts and suicidal ideations"
a) Make sure the Results are structured in a way that match the aims as set out in the Intro and Methods
Response: We have modified the review as a scoping review and changed our aim. The results are presented based on the aim.
''This scoping review aimed to map and to describe available epidemiological studies examining suicides, suicide attempts and suicidal ideations among children and young people exposed to war or armed conflicts. We also aimed to identify trauma exposures and risk and protective factors associated with the outcomes.'' (Introduction, Page 7)

2) I think it would be clearer to have separate headings for 'suicide deaths', 'suicide attempts', 'suicidal ideation'. Then, within each of these, explore 1) prevalence and 2) associated risk and protective factors
Response: We have revised the manuscript as scoping review thus; little emphasis was given on the prevalence and risk and protective factors of each outcome. We have, however provided a general overview of these aspect.

3) Also, you mention 'suicide plan' for the first time in the manuscript – it would be helpful to explain in the methods how this fits with the 3 outcomes that you aim to explore.

Response: To make the scoping review more focused on three outcomes suicide, suicide attempts and ideation, we decided to exclude suicidal plan assessed in the included studies (n=3). We revised the manuscript accordingly.

4) Suicide attempts and suicidal ideation and mental health is a confusing heading. See my suggestion in *Outcomes* 1a.
Response: We have made the changes in the revised manuscript.

Tables
• Some further structure could be added into the tables (it's unclear why they aren't in alphabetical order, too)
Response: As Table 1 includes geographical locations alphabetically and the studies were presented by publication date, with most recent studies listed first. The table was too large to be included in the manuscript, thus we made a new table presenting characteristics of the included studies. The previous table 1 is now included as supplementary materials (supplemental table 4).

o For table 2, under the three outcome sections/headings, could there be further sub-headings with
results organised by continent?
Response: We have updated the table 2 including sub-headings in the revised manuscript. The table is now included in the supplementary materials because it was too large to fit into the manuscript (see supplemental table 5).

Discussion
• In the first sentence, make sure you are clear about the purpose of this review. Specifically, "the
prevalence, risk and protective factors associated with suicides, attempts and suicidal ideations" – it's not clear in this first paragraph that you are looking at risk and protective factors.
Response: We have modified the review as a scoping review aiming to map available empirical evidence on suicidal thoughts and suicidal behaviors among young people exposed to war or armed conflicts.

• Differentiating between suicide rates during and post war is helpful.
Response: We have revised the manuscript accordingly.
"Four studies focused on suicides during and after the World War II. During the World War II, one study from England and Wales reported decrease in suicide rates [38] whereas a study from Scotland showed an increase, particularly among men. [31] During the Croatian Homeland War, a study reported increase in weapon-related suicide rates during the war. [33] In the Sri Lankan Civil War, suicide rates decreased during the war compared to pre-war levels. During the Bosnian War, one study showed decline in suicide rates eight years after the war, [35] while another study showed decrease during the war but an increase afterward. [34]
After the World War II, the suicide rates decreased in Japan followed by increase later. [39] In the United States, suicide rates remained stable for few years after the war before rising sharply. [32] Increases in weapon-related rates were reported after the Croatian Homeland

War. [33] ***Additionally, suicide rates were found to be higher among Balkan war migrants compared to non-war migrants.*** [37] ***The methods used for suicides across studies included hanging, firearms, sleeping pills and drowning.*** [34–36] **(Results, Page 12-13)**

• In general, try to structure based on your main aims (1. Prevalence; 2. Risk and protective factors) and will some structure when describing your results with reference to the literature based on the 3 outcomes. At times, it feels like there isn't a clear structure.
Response: We have revised the manuscript based on the aims.

• The section on limitations is too brief and warrants a paragraph of its own.
Response: We have added limitation in the revised manuscript.
''However, our review has several limitations. First, due to available resources within our research team, we only included English-language articles. War and armed conflicts occur across the world; therefore, it is possible that some publications may only be available in local languages. Second, given the difficulty of assessing suicidal behaviors after war or armed conflicts, most studies used convenient sampling and thus might lack representativeness. Third, the assessment tools were heterogeneous, limiting our ability to draw firm conclusions across studies and generalizability. Fourth, there is underrepresentation of some major conflict zones, namely Africa or the Middle East, where larger populations of children and young adults are affected by ongoing wars.'' (Page 18, Discussion)

• The discussion about future research is a sentence of the end of the Conclusion, which isn't enough and also warrants it's own section or to be integrated throughout the Discussion.
Response: We have incorporated need for future research in discussion section.
''In this review, refugees did not have an increased risk for suicides, suicide attempts or ideations. [37,56,72] ***…..There is need for further research in this population to understand the mechanisms contributing to risk and to explore protective factors from different countries.'' (Page 17, Discussion)***

''The mental health of children and young people in armed conflict regions is a dynamic process that requires an integrative ecological approach involving the family, school and community within their political, economic, and cultural contexts. [92] ***….. Regrettably the principle of protecting civilians from the horrors of war is often violated; nevertheless, researchers have an important role in developing empirically supported post-conflict recovery measures. Future research should identify mediating and moderating factors related to suicidality including cognitive, social and emotional factors, and investigate their interaction with broader mental health outcomes.''*** **(Page 19, Discussion)**

Reviewer: 2
Dr. Philippe Auby, CoBTeK lab
Comments to the Author:
Thank you very much for giving me the opportunity to review this interesting manuscript, and please find my comments below:

Abstract:
Would "More than half a billion…" be a more impactful wording than "Millions of children

and young people live in areas affected by war"?
According to UNICEF's Humanitarian Action for Children 2025, at least 460 million children are affected. In addition, as this report does not include youngsters between 18 and 24 years, the wording could be adjusted to provide a better sense of the magnitude of the issue.
https://www.unicef.org/reports/humanitarian-action-children-2025-overview
Response: We have modified the text in abstract section.
''Worldwide, billions of children and young people live in areas affected by war.''
(Abstract)

I believe you should further develop the 'Study Limitations' section.
-For instance, should at least a third, more specific limitation be added with respect to the underrepresentation of some major conflict zones, namely Africa or the Middle East, where larger populations of children and young adults are affected by ongoing wars?
Response: We have added limitation in the revised manuscript.
''Fourth, there is underrepresentation of some major conflict zones, namely Africa or the Middle East, where larger populations of children and young adults are affected by ongoing wars.'' (Page 19, Discussion)

-Should the first limitation you suggested and this potential third one also be considered as an opportunity to call for a future, larger "multilingual" review, and ultimately be mentioned in the discussion or conclusion?
Response: We have added text in the revised manuscript.
''Future larger multilingual review of the studies is required to provide comprehensive understanding of the empirical evidence of war on mental health of children and young people.'' (Page 20, Conclusion)

I wonder if the "other" reasons for excluding 7 studies should not be specified and reported, at the very least, on page 10 and possibly as a footnote to Figure 1.
Response: Thank you. We have modified the text and only reported reason for exclusion in the flow diagram, Figure 1.

The reviewed studies span a significant period. You mentioned on page 5 that historical factors can play a role in human reactions to catastrophes: have you found any hints that suicide risk among war-affected children and young people could vary across historical eras?
Response: Thank you for your comment. Indeed, suicide risk among war-affected children and young people may vary across historical eras, influenced by factors such as nature of the conflict, societal attitude toward mental health and availability of support systems. The recognition and understanding of the psychological trauma by war have evolved over time, potentially affecting reporting and addressing issues on suicidality.

I agree that the "results regarding refugees should hence be interpreted with caution ». Furthermore, as you pointed out, refugee studies are heterogeneous, conducted either in refugee camps close to the war zones or in third countries where people are seeking asylum. You might want to consider expanding the paragraph on page 16 about refugees and eventually explaining your rationale for including those studies. These studies might not be a perfect "proxy", however, they are informative.

Response: We have added text in the revise manuscript.
''The heterogeneity of refugee studies, conducted either in refugee camps, close to war zones or resettled in developed or developing countries and attitude of the host country may influence development of mental health of outcomes. Additionally, the length of stay in the host country has been suggested as a potential mediator, with refugees having lower risk initially, but after living in the new country for a longer period, the suicide risk has been reported to converge to the level of host population.[74] There is need for further research in this population to understand the mechanisms contributing to risk and to explore protective factors from different countries.'' **(Page 17, Discussion)**

Minor points:
-PTSD is never spelt out as Post Traumatic Stress Disorder. I would suggest doing this on either page 4 or 13.
Response: We have modified it in the revised manuscript.

-Define what a "convenient sampling" is.
Response: We used the term 'convenient sampling' in the text as the participants in most included studies were recruited randomly based on their accessibility. This approach is common in conflict settings, where conducting a representative sample is challenging or impossible due to security concerns, population displacement, and logistical barriers.
